# An integrated approach identifies the molecular underpinnings of murine anterior visceral endoderm migration

**DOI:** 10.1016/j.devcel.2024.05.014

**Published:** 2024-09-09

**Authors:** Shifaan Thowfeequ, Jonathan Fiorentino, Di Hu, Maria Solovey, Sharon Ruane, Maria Whitehead, Felix Zhou, Jonathan Godwin, Yentel Mateo-Otero, Bart Vanhaesebroeck, Antonio Scialdone, Shankar Srinivas

**Affiliations:** 1 Institute for Developmental and Regenerative Medicine, Department of Physiology, Anatomy and Genetics, University of Oxford, Old Road Campus, Roosevelt Drive, Oxford OX3 7TY, UK; 2 Institute of Epigenetics and Stem Cells, Helmholtz Zentrum München, German Research Center for Environmental Health, Munich 81377, Germany; 3 Institute of Functional Epigenetics, Helmholtz Zentrum München, German Research Center for Environmental Health, Neuherberg 85764, Germany; 4 Institute of Computational Biology, Helmholtz Zentrum München, German Research Center for Environmental Health, Neuherberg 85764, Germany; 5 Center for Life Nano- & Neuro-Science, Istituto Italiano di Tecnologia, Rome 00161, Italy; 6 UCL Cancer Institute, University College London, London WC1E 6DD, UK; 7 University of Texas Southwestern Medical Center, Dallas, TX 75390, USA; 8 Unit of Cell Biology, Department of Biology, University of Girona, Girona 17004, Spain

**Keywords:** anterior visceral endoderm, embryonic patterning, mouse embryogenesis, cell migration, single-cell transcriptomics, phosphoproteomics, semaphorin signaling

## Abstract

The anterior visceral endoderm (AVE) differs from the surrounding visceral endoderm (VE) in its migratory behavior and ability to restrict primitive streak formation to the opposite side of the mouse embryo. To characterize the molecular bases for the unique properties of the AVE, we combined single-cell RNA sequencing of the VE prior to and during AVE migration with phosphoproteomics, high-resolution live-imaging, and short-term lineage labeling and intervention. This identified the transient nature of the AVE with attenuation of “anteriorizing” gene expression as cells migrate and the emergence of heterogeneities in transcriptional states relative to the AVE’s position. Using cell communication analysis, we identified the requirement of semaphorin signaling for normal AVE migration. Lattice light-sheet microscopy showed that *Sema6D* mutants have abnormalities in basal projections and migration speed. These findings point to a tight coupling between transcriptional state and position of the AVE and identify molecular controllers of AVE migration.

## Highlights

• The AVE is composed of transcriptionally and spatially distinct sub-populations
• The AVE transcriptional state is downregulated upon its lateral displacement
• Cytoskeletal proteins and modulators are subjected to AVE-specific modifications
• Semaphorin 6D-mediated signaling is necessary for proper AVE migration

## Graphical abstract

**Figure:**
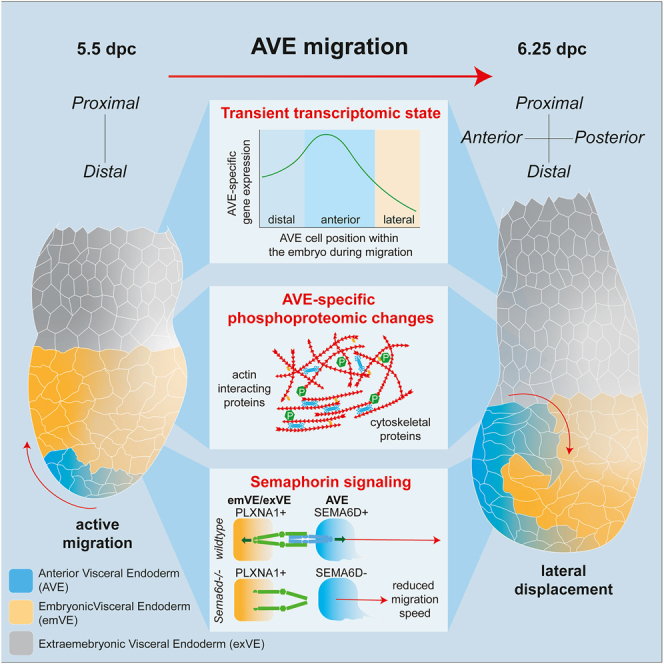

## Teaser

In this study, Thowfeequ and Fiorentino et al. interrogate the molecular underpinnings of AVE migratory behavior that initiate anterior-posterior patterning of the developing mouse embryo. They outline the changing transcriptional state of migrating AVE cells, identify its phosphoproteomic makeup, and show a semaphorin-plexin signaling requirement for its proper migration.

## Introduction

Axial specification is a relatively late event during mammalian embryogenesis, as extra-embryonic tissues essential for this process need to be established first. One such tissue is the visceral endoderm (VE). The mouse VE, equivalent to the hypoblast in other amniotes, is a simple epithelium encapsulating the pluripotent epiblast (Epi) and the extra-embryonic ectoderm (ExE).^1^^,^^2^^,^^3^ Around 5.5 days post coitum (dpc), a sub-region of the embryonic VE, termed the anterior visceral endoderm (AVE; also called the distal VE at this stage based on its initial position at the distal tip of the egg cylinder) is specified.^4^^,^^5^ AVE cells have a distinct columnar morphology^6^^,^^7^ and expression of specific markers including *Hhex*, *Lhx1*, *Otx2*, *Lefty1*, and *Cer1*.^5^^,^^8^

In response to unknown cues, AVE cells initiate a characteristic unidirectional migration toward the presumptive anterior region of the embryo, and over just 4–5 h, they reach the boundary between the Epi and ExE,^7^ which represents an endpoint to their proximal migration. From this position, AVE cells secrete inhibitors of the Wnt and Nodal signaling pathways (DKK1, CER1, and LEFTY1), thereby restricting primitive streak (PS) formation to the opposite side of the egg cylinder^9^^,^^10^^,^^11^^,^^12^^,^^13^ and establishing the earliest features of anterior-posterior (AP) polarity. Cells from the distal tip of the egg cylinder continue to migrate to the presumptive anterior so that by 6.25 dpc, the original AVE cells to occupy the anterior position have been displaced laterally and replaced by later-arriving AVE cells.^7^^,^^14^^,^^15^

The directional migration of AVE from the distal tip of the egg cylinder to the boundary with the ExE resolves an “AP” axis that is initially aligned with the proximal-distal (PD) axis of the egg cylinder to a definitive one that is orthogonal to it.^16^ While VE transcriptional profiles are available,^17^^,^^18^^,^^19^ a comprehensive set of testable predictions on their dynamics and functional roles and the experimental validations of computational findings about the origin, fate, and spatial heterogeneities of the AVE are lacking.

Mutants in which the AVE cells fail to migrate show defects in the localized formation of the PS.^5^ However, with a total arrest of AVE migration, such mutants are inadequate for studying the intricate cellular mechanism by which the AVE’s unique migratory behavior is controlled. AVE cells need to negotiate their way through the surrounding VE monolayer, which maintains epithelial integrity.^20^ AVE cells show characteristics of active migration, such as polarized cellular projections^7^ arising from their basal aspect.^14^ Therefore, to facilitate their migration, AVE cells must coordinate two very different sets of behavior: remodeling junctions apically and sending out polarized projections basolaterally. The actomyosin cytoskeleton plays a central role during AVE migration^14^^,^^21^^,^^22^^,^^23^ and is important for basal projections. However, the specific role of these protrusions in migration and the signals that regulate them remain unknown.

In this study, we combined full-length, high-coverage single-cell RNA sequencing (scRNA-seq) and bulk phosphoproteomics with high-resolution imaging of the VE prior to and during AVE migration to characterize differences between AVE cells and surrounding VE cells. By doing so, we defined the transient transcriptional state of AVE cells during their emergence and migration and visualized these changes relative to changes within the rest of the embryo. We established the “fate” of AVE cells once they had completed migration and validated these findings using lineage labeling. Finally, we identified signaling pathways likely to play important roles during AVE migration. Using computational cellular motion phenotype analysis on lattice light-sheet live-imaging data of AVE migration in *Sema6d* knockout (KO) embryos, we identify a requirement of semaphorin signaling in fine-tuning AVE basal projections, thereby controlling the progression of migration.

## Results

### Single-cell transcriptomic profiling identifies VE sub-populations spatially organized along an emergent AP axis

**Figure 1:**
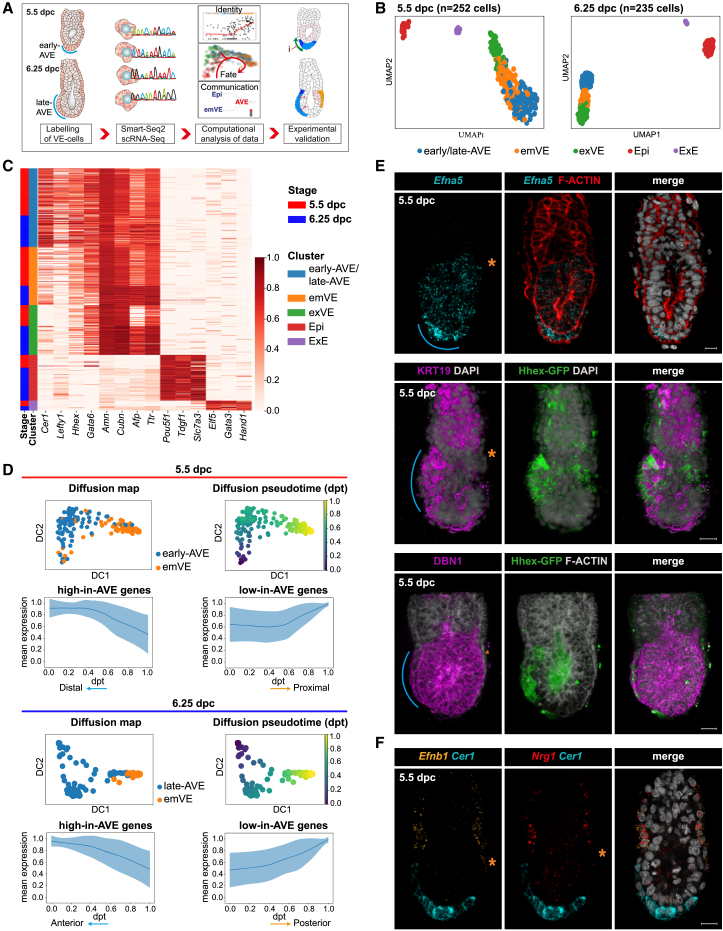
Identification and validation of anterior and posterior VE markers (A) Schematic summarizing the isolation and the transcriptional characterization of VE cells. (B) Uniform manifold approximation and projection (UMAP) plots of cells from 5.5-dpc (*n* = 40) and 6.25-dpc (*n* = 11) embryos, clustered into five groups at each stage: early- or late-anterior visceral endoderm (AVE), rest of the VE surrounding the epiblast or ExE (emVE and exVE, respectively), the epiblast (Epi), the extra-embryonic ectoderm (ExE). (C) Heatmap of normalized log expression levels of known marker genes for all identified cell types. (D) First two diffusion components (DC1 and DC2) of early- or late-AVE and emVE cells, colored according to cluster (left) and diffusion pseudotime (dpt) coordinate (right). Mean standardized expression of the genes belonging to high-in-AVE and low-in-AVE groups. (E) Spatial expression of selected high-in-AVE markers, using HCR (*Efna5*) or immunofluorescence (KRT19 and DBN1). Hhex-GFP marks the AVE. (F) Spatial expression of selected low-in-AVE markers (*Efnb1* and *Nrg1*). *Cer1* expression marks the AVE. In (E) and (F), blue lines indicate the position of the AVE, and the orange asterisk marks the posterior. Scale bars represent 20 μm, and embryos are orientated anterior to the left. See also Tables S1, S2, S3, S4, and S13.

To sample cells at various stages of AVE migration from start to finish, we collected single cells from multiple litters at 5.5 and 6.25 dpc, enriching for VE cells (STAR Methods). Cells from disaggregated embryos were isolated using fluorescence-activated cell sorting (FACS) and processed through the Smart-seq2 protocol, which allows for full-length, high-coverage scRNA-seq^24^ (Figure 1A). After quality control, 252 cells at 5.5 dpc and 235 at 6.25 dpc were retained for downstream analysis. We performed unsupervised clustering (STAR Methods), which identified five clusters per stage (Figure 1B; STAR Methods). The two smallest clusters corresponded to Epi and ExE, based on the expression pattern of known marker genes (Figure 1C). The VE enrichment was successful as the remaining three larger clusters at each stage represented VE sub-types (expressing *Gata6* and *Amn*), which we further annotated as early- or late-AVE (at 5.5 and 6.25 dpc, respectively, expressing high levels of *Cer1*, *Lefty1*, and *Hhex*), “embryonic VE”-non-migratory VE cells (emVE; overlying the Epi, with lower expression of *Cer1, Lefty1*, and *Hhex*), and “extra-embryonic VE”-VE cells (exVE; overlying the ExE, with the highest *Cubn* and low *Afp* expression; Figures 1C, S1A, and S1B). Additionally, we unbiasedly identified marker genes of each cluster (Figure S1A; Tables S1 and S2).

To understand how the transcriptional profiles of the VE sub-types related to each other, we analyzed the relative distance and the connectivity between them with partition-based graph abstraction (PAGA^25^). This showed that VE clusters were highly connected with the AVE at both stages, being most closely connected to the respective emVE (Figures S1C and S1D). Moreover, AVE is transcriptionally more distinct from the surrounding emVE at 6.25 dpc than at 5.5 dpc.

Given the subtle differences between the transcriptomes of the AVE and emVE, we ordered the cells from both stages through the definition of a diffusion pseudotime (dpt) coordinate^26^^,^^27^ originating from the respective AVE cluster at each stage (Figure 1D). We identified the genes differentially expressed in pseudotime, and using an unsupervised clustering analysis (STAR Methods), we split them into two gene groups based on their expression in the AVE cell clusters (“high-in-AVE” and “low-in-AVE” gene groups; Figure 1D; Tables S3 and S4). The presence of well-known AVE markers among the high-in-AVE genes (e.g., *Cer1* and *Hhex*; Figure S1E) suggests that the pseudotime axis (dpt) tracks the spatial position of cells along the AP axis at 6.25 dpc and the PD axis at 5.5 dpc.

To test this hypothesis, we chose several genes from the high-in-AVE and low-in-AVE groups common to both stages to determine their expression patterns in embryos via multiplexed *in situ* hybridization chain reaction (HCR) and whole-mount immunofluorescence (Figures 1E, 1F, S1A, S1F, and S2A–S2C). AVE cells were independently identified based either on their distinct columnar morphology or the expression of reference AVE markers such as *Cer1* or the Hhex-GFP transgene.^28^ A selection of high-in-AVE genes encoding proteins with potential links to cell migration were chosen for further validation. *Efna5* transcript and KRT19 and DBN1 proteins were all expressed at high levels in AVE cells at 5.5 (Figure 1E) and 6.5 (Figures S2A and S2C) dpc with little expression in surrounding emVE cells, confirming that the dpt coordinates indeed represented the PD and AP axes of the 5.5- and 6.25-dpc embryos, respectively. Additionally, KRT19 and DBN1 were also expressed in the ExE and the Epi, respectively, in agreement with the scRNA-seq results (Figures 1E,S1A, S2A, and S2B).

Next, we validated the expression of two low-in-AVE genes, *Nrg1* and *Efnb1* (Figures S1A and S1F), showing that their primarily exVE expression domains extended distally into the emVE on the presumptive-posterior side opposite to the AVE, at 5.5 and 6.5 dpc (Figures 1F and 5H). Overall, our statistical analysis and the experimental validation of selected AVE marker genes highlight a distinction between AVE and emVE cells already at 5.5 dpc and corroborate the interpretation of the dpt axis as a spatial axis, spanning the emergent AP axis.

Finally, we performed isoform analysis (STAR Methods) to identify the differential expression of splice variants between different cell types. This highlighted several differences, such as *Rin3* belonging to a family of proteins that are regulators of epithelial cell adhesion and migration,^29^ which showed uniform expression among VE cells but with protein-coding isoforms only enriched within the AVE cells (Figure S1G; Table S13), indicating possible AVE-specific heterogeneities to the proteome that might not be reflected in the transcriptome alone.

### Leveraging single-cell transcriptomics data to explore the proteomic and phosphoproteomic landscape of the VE

**Figure 2:**
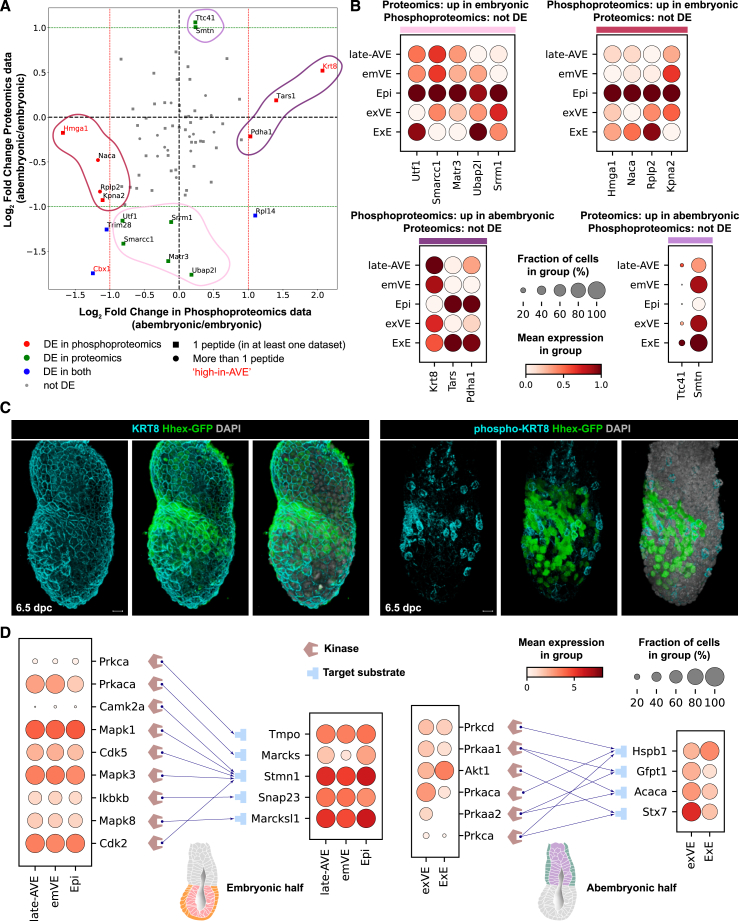
Proteomic and phosphoproteomic landscape of the post-implantation/pre-gastrulation embryo (A) Scatterplot of log_2_ fold change between the embryonic and abembryonic halves, in proteomics vs. phosphoproteomics data. Each marker represents a protein, and those corresponding to genes from the high-in-AVE group are highlighted in red. Pearson’s correlation coefficient = 0.36; *p* = 1.3 × 10^−3^. (B) Dot plots of gene expression (scRNA-seq) corresponding to (phospho)proteins that are differentially expressed between the embryonic and abembryonic halves only in one dataset (sets outlined in A). (C) Surface renderings, showing expression of KRT8 (*n* = 7) and phospho(Ser23)-KRT8 (*n* = 5) visualized by immunofluorescence in 6.5-dpc embryos. Hhex-GFP marks the AVE. Scale bars represent 20 μm. (D) Kinase-substrate bipartite networks predicted for substrate proteins upregulated in the embryonic (left) or abembryonic (right) half in the phosphoproteomics dataset. Dot plots of gene expression (scRNA-seq) are shown for the corresponding kinases and substrates. See also Tables S5, S6, S7, S8, S14, S15, S16, and S17.

To determine the extent to which the transcriptional profile of a cell might reflect its functional protein complement, we compared our single-cell transcriptomics data with mass spectrometry-driven bulk proteomics and phosphoproteomics data from 6.25- to 6.5-dpc embryos, bisected into “embryonic” (Epi and overlying emVE) and “abembryonic” (ExE and overlying exVE) portions to retain some spatial information (Figure S1B). Differentially expressed or phosphorylated proteins between the embryonic and abembryonic halves were identified (Figures 2A, S2D, and S2E; Tables S5, S6, S7, and S8; STAR Methods).

A functional enrichment analysis of proteins differentially expressed or phosphorylated between the two halves (Figure S2G; Tables S16 and S17; STAR Methods) identified several expected enriched terms. These included, terms associated with pluripotency of the Epi and its maintenance, for the embryonic half, and significant terms related to secretory and absorptive functions, for the abembryonic half which contributes to the placenta (Figure S2G). This indicates that the (phospho)proteomics data are a reliable reflection of the functional characteristics of the collected tissues.

Despite the smaller number of proteins quantified in the (phospho)proteomics, compared with the transcripts detected in scRNA-seq, overall, there was good agreement between the datasets, as shown by the statistically significant correlation between the log_2_ fold changes computed from them (STAR Methods; Figures S2D and S2E). We found a few differences, which could be accounted for by the differential expression of splice variants between different cell types (see previous section). Additionally, we also identified proteins such as KMT2A and PML, important in cell migration,^30^^,^^31^ which showed differences in phosphorylation but were transcriptionally expressed uniformly across the VE (Figure S2E).

Comparison of proteomics and phosphoproteomics data can help identify proteins that might carry out their function via posttranslational regulation. Hence, we identified proteins that were equally abundant, but differentially phosphorylated, across the two halves (Figures 2A and 2B). KRT8 was one such protein, which was equally abundant in the two halves but highly phosphorylated in the abembryonic half (Figure 2A).

Since the embryonic and abembryonic halves each contained Epi and ExE cells, respectively, along with VE cells constituting a comparatively small proportion of the cellular mass, it is possible that differential phosphorylation specific to sub-populations of the VE might be masked by those specific to the Epi or ExE. Therefore, we leveraged our scRNA-seq data to help identify interesting candidates from the phosphoproteomic data specific to the AVE (Figure 2A). Our scRNA-seq data showed that the transcript for *Krt8* were elevated in the AVE compared with other VE populations (Figure 2B), consistent with elevated levels of KRT8 protein within the AVE compared with the remaining VE.^32^ Using phospho-specific antibodies we showed that among the VE cells, Ser23-phosphorylated KRT8 was primarily localized to the AVE population, compared with more widespread expression of the total protein (Figure 2C; *n* = 7 and 5).

We next focused on high-in-AVE genes that were associated with cell migration, whose associated proteins show an upregulation in the phosphoproteomic data from the embryonic halves (e.g., *Dbn1*, *Marcks*, *Marcksl1*, and *Stmn1*; Figures S2A, S2E, and S2F; El Amri et al.,^33^ Ni et al.,^34^ and Tanabe et al.^35^). Immunofluorescence and HCR validation for MARCKS and MARCKSL1 suggested that they might be subjected to posttranslational regulation in a tissue-specific manner to achieve finer control over AVE migration (Figures S2A, S2E, and S2F)

Finally, to make inferences relating to signaling interactions between these key tissues, we inferred putative kinase-substrate interactions for the differentially phosphorylated proteins between the two halves, represented as two bipartite networks (Figure 2D; STAR Methods). This gives us an indication of the tissue-specific players that are responsible for specific posttranslational modifications. Examples include the kinases *Prkaca* and *Mapk8*, which are expressed in the late-AVE at the RNA level (Figure 2D) and are known to phosphorylate MARCKS and MARCKSL1, respectively, in the context of cell migration.^36^^,^^37^^,^^38^ Taken together, these analyses illustrate the utility of phosphoproteomics in complementing our scRNA-seq data for the identification of candidates important in AVE migration.

### AP asymmetries across the entire embryo is predicated on anterior-specific gene expression within the VE

**Figure 3:**
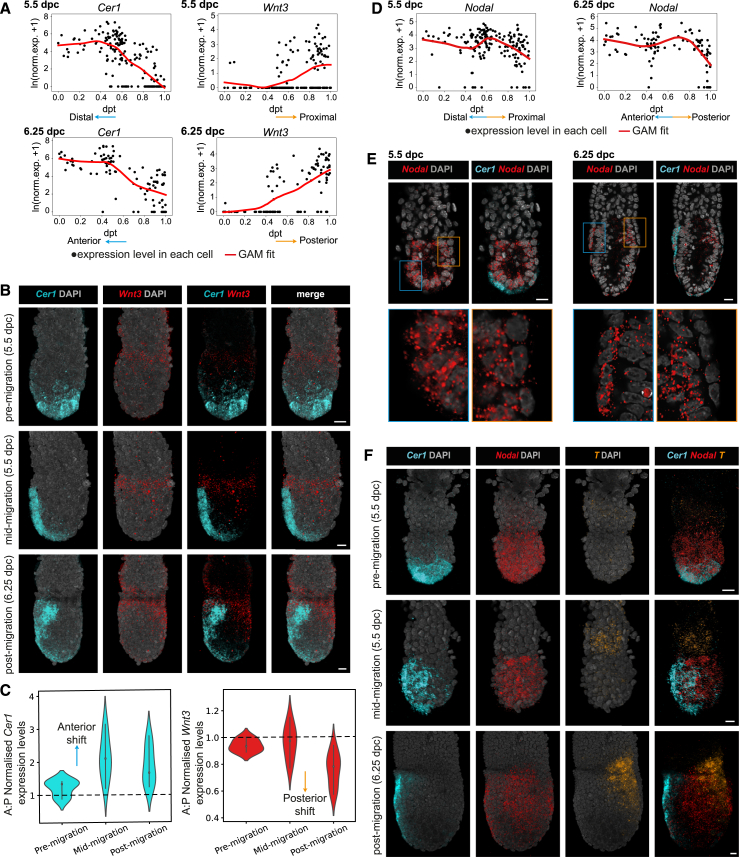
Symmetry breaking along the anterior-posterior axis in direct comparison to AVE migration (A) Expression patterns of *Cer1* and *Wnt3* in the AVE and emVE clusters, as a function of diffusion pseudotime. (B) Volume renderings showing changes to the *Wnt3* expression domain (visualized by HCR) relative to *Cer1-*expressing AVE cells, in pre-migration (*n* = 6), mid-migration (*n* = 16), and post-migration (*n* = 27) embryos. (C) Quantification of the VE *Cer1* and *Wnt3* HCR signals in the anterior and posterior regions of pre- (*n* = 6), mid- (*n* = 8), and post-migration (*n* = 9) embryos. Expression levels are presented as an anterior:posterior (A:P) ratio, with 1 indicating balanced expression of the two markers. Data are represented as mean ± SEM. (D) Expression patterns of *Nodal* in AVE and emVE clusters, as a function of diffusion pseudotime. (E) Optical sections through embryos showing the distinct expression of *Nodal* in the epiblast and the VE relative to that of *Cer1* at 5.5 (*n* = 6) and 6.25 (*n* = 4) dpc. Magnifications of the boxed anterior and posterior regions are shown underneath to highlight distinct *Nodal* expression in VE and epiblast. (F) Volume renderings showing changes in *Nodal* and *T* expression relative to the position of *Cer1-*expressing AVE cells in pre-migration (*n* = 3), mid-migration (*n* = 6), and post-migration (*n* = 3) embryos. In (A) and (D), each black dot represents a cell; the red line shows the fit obtained from a generalized additive model (GAM). In (B), (E), and (F), scale bars represent 20 μm; and embryos are orientated anterior to the left. See also Videos S1, S2, S3, and S4.

Our dpt captures the transformation of a PD asymmetry in marker expression into an AP asymmetry. We therefore used it to investigate the sequence in which asymmetric gene expression emerges first within the VE itself and then in comparison with the Epi. Our dpt analyses showed that at both 5.5 and 6.25 dpc, anterior-specific asymmetric expression of *Cer1* is more pronounced and robust, compared with the posterior-specific asymmetric expression of *Wnt3*, previously suggested as an early posterior VE marker^39^ (Figure 3A). We experimentally validated this using multiplexed HCR to detect both *Cer1* (distal/anterior) and *Wnt3* (proximal/posterior) simultaneously in the same embryos (Figures 3B and 3C). Such multiplexing is important in mitigating against natural variations across embryos from a single developmental time point (Figures S3A and S3B) at a stage when transcriptional, positional, and cellular changes occur rapidly. We found that despite these variations, in pre-migration 5.5-dpc embryos, *Cer1* was expressed symmetrically at the distal tip (*n* = 6), and at mid-migration, *Cer1* started to show clear asymmetric localization to the prospective anterior (*n* = 16). *Wnt3* was expressed symmetrically across the proximal egg cylinder (both Epi and emVE) at these stages. We only observed a complete segregation of the *Wnt3* expression domain to the presumptive posterior in embryos staged beyond 6.0 dpc, before the PS had emerged, but after the *Cer1*-expressing AVE cells had reached the Epi-ExE boundary (*n* = 27; “post-migration”; Figures 3B and 3C; Video S1). This indicates that an axial pattern emerges first by the asymmetric expression of anterior markers within the VE, followed by asymmetric expression of posterior markers within the VE and Epi.

Video S1. Expression of *Cer1* and *Wnt3* in a mid-migration 5.5 dpc (E5.5) embryo, related to Figure 3 The expression domain of *Cer1* is shifted toward the anterior of the embryo, while the expression of *Wnt3*, although enriched in the posterior, still extends across the entire proximal half of the egg cylinder. DAPI marks the nuclei.

Next, we tested if DKK1 might act as a guidance cue for AVE cells.^12^ The dpt analysis showed that unlike *Cer1*, which marked the distal and anterior regions of VE before and after migration at 5.5 and 6.25 dpc, respectively, *Dkk1* became elevated in the anterior, only at 6.25 dpc (Figure S3C). In agreement with this and previous reports on DKK1 protein expression,^8^ our multiplexed HCR experiments showed that *Dkk1* expression only became anterior specific after AVE migration was well underway (*n* = 4; Figure S3E, post-migration). Prior to this, *Dkk1* was expressed in cells interspersed throughout the distal region (*n* = 9; “pre-migration”) or restricted to distinct domains in the anterior and posterior (*n* = 10; Figure S3E, “mid-migration”; Video S2), arguing against a role for DKK1 in AP symmetry breaking and guiding AVE migration.

Video S2. Expression of *Cer1* and *Dkk1* in a mid-migration 5.5 dpc (E5.5) embryo, related to Figures 3 and 5 The expression of *Cer1* is confined to the AVE in the anterior portion of the embryo, while the expression of *Dkk1* is seen as two distinct domains ahead of and behind the *Cer1*-expressing cells, respectively in the anterior (A) and posterior (P) regions of the egg cylinder. DAPI marks the nuclei.

Axial asymmetry generated by directional AVE migration is transferred to the Epi by the AVE inhibiting the expression of PS-specific genes in the subjacent Epi. To determine the precise sequence of asymmetric marker expression in the VE relative to the Epi, we computationally analyzed the temporal expression patterns of the posterior marker *Nodal* and the reference PS marker, *Brachyury/T*,^40^^,^^41^ in direct relation to the AVE marker *Cer1*, followed by experimental validation. Our dpt analyses identified that at 6.25 dpc, *Nodal* was significantly depleted in the posterior VE, starting off from a decreasing trend toward the proximal end at 5.5 dpc (Figure 3D). This corresponded to high expression of *Nodal* in *Cer1-*positive AVE cells at 5.5 and 6.25 dpc (*n* = 6 and 4; Figure 3E), consistent with *Nodal* being important for the initial induction of the AVE.^42^ Within the Epi, as expected, *Nodal* was detected uniformly throughout in pre-migration and mid-migration embryos (*n* = 3 and 6; Figure 3F; Video S3) and only became restricted to the posterior Epi after *Cer1*-expressing cells were already positioned along the anterior and lateral sides of the egg cylinder (*n* = 3, Figure 3F, post-migration; Video S4). *T* was detected in only a limited number of Epi cells, with visible expression first in the ExE^39^ followed by posterior-specific Epi expression only at 6.5 dpc (Figure 3F; Video S4). We did not detect significant *T* expression in any VE population at any stage (Figures S3D and S3F). Collectively, these results establish a sequence where robust axial pattern in the Epi only emerges at 6.25 dpc, approximately 12–18 h after such asymmetry is seen in the VE at 5.5 dpc as a result of AVE migration.

Video S3. Expression of *Cer1, T*, and *Nodal* in a mid-migration 5.5 dpc (E5.5) embryo, related to Figure 3 The expression of *Cer1* (cyan) is confined to the AVE in the anterior portion of the embryo, while the expression of *T* (yellow) is seen only in the ExE and that of *Nodal* (red) is spread throughout epiblast. DAPI (gray) marks the nuclei.

Video S4. Expression of *Cer1, T*, and *Nodal* in a post-migration 6.25 dpc (E6.25) embryo, related to Figure 3 The expression of *Cer1* is confined to the AVE in the anterior portion of the embryo, while the expression of *T* and *Nodal* is enriched in the posterior half of the egg cylinder. DAPI (gray) marks the nuclei.

### The AVE is a transient state associated with a spatially and temporally heterogeneous cell population

**Figure 4:**
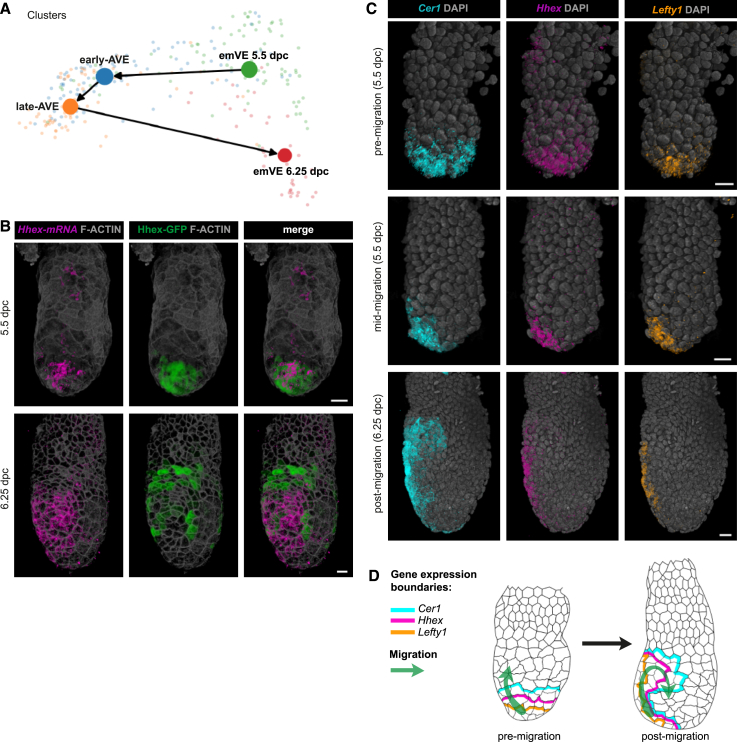
Origin and fate of the AVE (A) PAGA graph computed from RNA velocities and projected on the first two diffusion components of a diffusion map of the AVE and emVE clusters, combining the data from both stages. (B) Direct comparison of short-term lineage-labeled AVE cells (expressing the Hhex-GFP reporter) and the contemporaneous expression of the endogenous *Hhex* transcript (visualized by HCR) in 5.5-dpc (*n* = 7) and 6.25-dpc (*n* = 5) embryos. Embryos are rotated by ∼20° about their PD axis to show the full anterior surface along with lateral sides. (C) Volume renderings directly comparing changes to the expression domains of the AVE markers *Cer1*, *Hhex*, and *Lefty1* in embryos before (*n* = 13), during (*n* = 18), and after (*n* = 8) AVE migration. Embryos are orientated anterior to the left. (D) Schematic summarizing changes to *Cer1*, *Hhex*, and *Lefty1* expression domains throughout the VE because of AVE migration. Scale bars represent 20 μm. See also Videos S5 and S6.

Next, we compared AVE cells from both stages to understand the transcriptional changes within the AVE as it matures and starts patterning the underlying Epi. We used RNA velocity analysis^43^ with the scVelo implementation,^44^ comparing the ratio between spliced and unspliced mRNA, to estimate the transcriptional dynamics of differentiating cell populations (STAR Methods). The velocities of the single cells were projected onto the first two diffusion components of a diffusion map of AVE and emVE cells, computed by integrating the two stages (STAR Methods; Figure S4A). The stream indicates a trajectory originating from the 5.5-dpc emVE, transitioning *through* the 5.5-dpc AVE, then the 6.25-dpc AVE, and ending in the 6.25-dpc emVE (Figure 4A). This suggested the hypothesis that upon induction at 5.5 dpc, AVE cells (equivalent to the DVE) emerge as a transcriptionally distinct population from the emVE, becoming transcriptionally more divergent from it as they migrate anteriorly and “mature,” but transition back toward an emVE state as they are displaced laterally by the following stream of cells that give rise to the AVE at 6.25 dpc.

We performed a short-term lineage labeling experiment to test this hypothesis, using the well-established Hhex-GFP line^28^ that labels AVE cells. We took advantage of the perdurance of GFP to label cells that expressed *Hhex* in the past, combined with HCR to assay the current transcriptional state based on the presence or absence of the endogenous *Hhex* transcript (Figure 4B). At 5.5 dpc, *Hhex* mRNA and Hhex-GFP fluorescent signal both co-localized in migrating AVE cells at the distal end of the egg cylinder. However, by 6.25 dpc, in embryos in which migrating AVE cells had reached the boundary and were being displaced laterally, most proximal and lateral Hhex-GFP cells (that is, the earliest DVE/AVE cells that were induced at 5.5 dpc) were no longer expressing endogenous *Hhex* transcript (Figure 4B; Video S5). This was consistent with the RNA velocity predictions and highlights the transient nature of the AVE state, whereby VE cells acquire markers characteristic of the AVE and then lose them as they revert to an emVE state once they have migrated anteriorly and started to be displaced laterally.

Video S5. Short-term lineage tracing of AVE cells, related to Figure 4 Cells expressing the *Hhex* transcript extend all the way to the distal tip of the embryo, while those cells in the process of downregulating endogenous *Hhex* expression but still marked by Hhex-GFP occupy the most proximal and lateral sides of the embryo. F-actin marks cell boundaries.

We next looked at the expression of other AVE markers (*Cer1* and *Lefty1*), along with *Hhex*, in the same embryos at different stages of migration. We found that before migration is initiated, all markers were restricted to the distal tip with the expression of *Cer1* extending proximally over a larger region, followed by *Hhex*, then *Lefty1* (*n* = 13; Figure 4C pre-migration; Video S6). By mid-migration, we detected a discernible asymmetry in the expression domains of all three transcripts (*n* = 18). At post-migration stages, while the domain of expression of *Lefty1* and *Hhex* never extended laterally, *Cer1* was detected at relatively low levels in lateral cells (*n* = 8), consistent with a successive loss of AVE identity and a gradual shift back toward an emVE state upon lateral displacement (Figure 4D).

**Figure 5:**
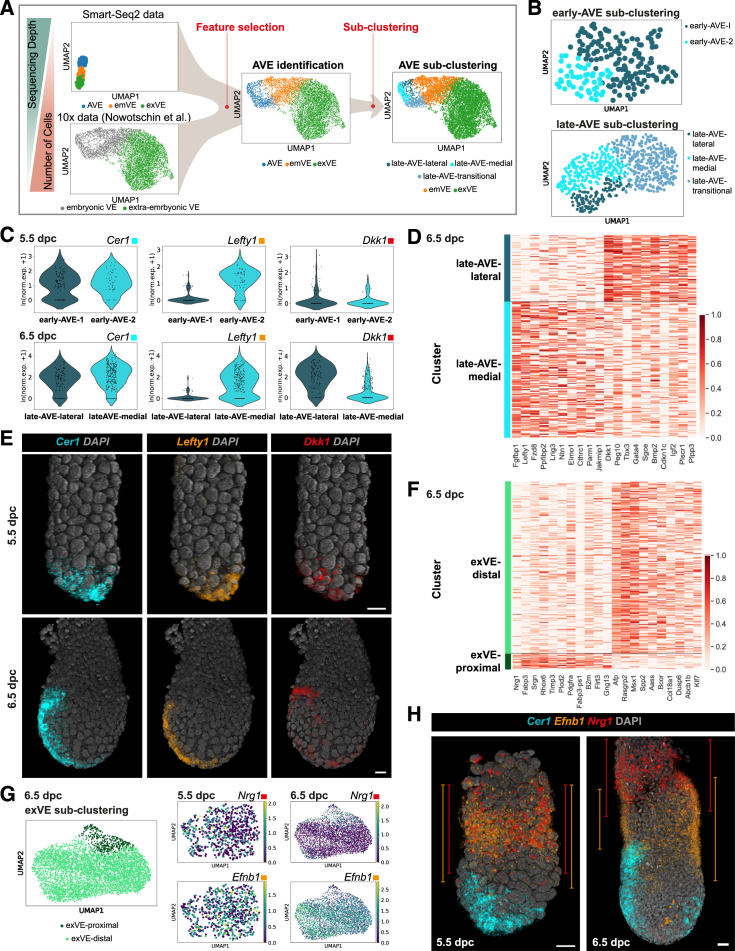
Spatial mapping of the transcriptional heterogeneity seen within the AVE and exVE clusters (A) Computational strategy used for VE sub-clustering of 10× scRNA-seq data (Nowotschin et al.^17^), using gene expression features extracted from our Smart-seq2 data. The AVE cluster was selected for further sub-clustering analysis. (B) UMAP plots of cells (data from Nowotschin et al.^17^) belonging to the AVE at 5.5 dpc (top) and 6.5 dpc (bottom), colored according to sub-clusters (Figure S4 and STAR Methods for AVE identification in these data). (C) Violin plots showing *Cer1*, *Lefty1*, and *Dkk1* normalized log expression in cells grouped according to the clusters in (B), for both stages. (D) Heatmap showing normalized log expression of the top genes upregulated in the late-AVE-medial cluster and the AVE-lateral cluster (10 each), obtained through a differential expression analysis between the two clusters at 6.5 dpc. (E) Volume renderings showing the expression domains of *Cer1, Lefty1*, and *Dkk1*, used to distinguish between the sub-clusters of the AVE at 5.5 (*n* = 8) and 6.5 (*n* = 3) dpc. (F) Heatmap showing normalized log expression of the top 10 genes upregulated in the exVE-proximal cluster and the exVE-distal cluster at 6.5 dpc. *Nrg1* (which ranked 13^th^ in the exVE-proximal cluster by adjusted *p* value) was manually added to the list. (G) UMAP plots showing sub-clusters within the exVE at 6.5 dpc (left) and of cells belonging to the exVE cluster at 5.5 (center) and 6.5 dpc (right), colored according to normalized log expression of *Nrg1* and *Efnb1*. (H) Volume renderings showing changes to the expression domains of *Efnb1* and *Nrg1* between 5.5 (*n* = 6) and 6.5 (*n* = 4) dpc in comparison with *Cer1-*expressing AVE cells. Orange and red lines represent, for *Efnb1* and *Nrg1*, respectively, the proximal-to-distal extent of their expression domains. Scale bars represent 20 μm, and embryos are orientated anterior to the left. See also Tables S9, S10, S11, and S12 and Videos S2 and S6.

Video S6. Expression of *Cer1, Hhex*, and *Lefty1* in a pre-migration 5.5 dpc (E5.5) embryo, related to Figures 4 and 5 The expression of all three transcripts is localized to the distal tip of the egg cylinder with the expression domain of *Cer1* (cyan) extending the widest and most proximal, followed by that of *Hhex* (magenta), and then *Lefty1* (yellow). DAPI (gray) marks the nuclei.

The heterogeneities in expression of these archetypal AVE markers in relation to the spatial position of cells (Figures 1C and 4C) suggested that transcriptionally and spatially distinct sub-populations exist even within the relatively small group of AVE cells. To uncover the molecular signatures of these AVE sub-populations, we took advantage of the large numbers of cells from a published 10× scRNA-seq dataset,^17^ which includes 1,067 and 7,182 cells from 5.5- and 6.5-dpc embryos, respectively, annotated as embryonic or extra-embryonic VE. We leveraged the higher sequencing depth of our Smart-seq2 dataset to accurately define AVE transcriptional signatures among VE cell types, and we used the large number of cells in the 10× dataset, despite the shallower sequencing depth, to identify AVE sub-clusters with greater confidence (STAR Methods; Figures 5A, S4B, and S4C).

Three AVE sub-clusters emerged at 6.5 dpc from the 10× dataset (Figure 5B, “late-AVE sub-clustering”; Table S9). One of these clusters, despite having low levels of several canonical AVE markers including *Cer1*, *Hhex*, and *Lefty1* (Figure S4D), in comparison with emVE cells, showed relatively higher levels of the high-in-AVE genes we identified previously (Figures 1D and S4E; Table S10). This cluster, therefore, likely represented the transitional state (late-AVE-transitional) occupied by cells that originated as the early-AVE at 5.5 dpc and that were in the process of downregulating the AVE transcriptional program to acquire an emVE-like transcriptional state. This was also consistent with our HCR results for expression of *Cer1*, *Hhex*, and *Lefty1* and our AVE fate analysis (Figures 4B and 4C). The two other sub-clusters both had high *Cer1*-expression and were distinguished by the expression of *Lefty1* in one and *Dkk1* in the other (Figure 5C) along with several differentially expressed genes (Figure 5D; Table S11). Using *Cer1*, *Lefty1*, and *Dkk1* to provide spatial landmarks, we mapped these two sub-clusters of the late-AVE onto the medial and lateral regions of the 6.5-dpc embryo, respectively (Figure 5E, “6.5 dpc”).

We next performed a sub-clustering of 5.5-dpc AVE cells from the 10× dataset, employing the top 20 genes differentially expressed between the AVE-medial and -lateral sub-clusters at 6.5 dpc (Figure 5B, “early-AVE sub-clustering”; STAR Methods). This identified two 5.5-dpc AVE sub-clusters with comparable levels of *Cer1* but different levels of *Lefty1* (Figure 5C), spatially segregated at 5.5 dpc with the *Cer1*+, *Lefty1*− cells (early-AVE-1) population more proximal to the *Cer1*+, *Lefty1*+ population (early-AVE-2) located at the very distal tip (Figure 5E, “5.5 dpc”). However, the transcriptional differences between these two sub-clusters are very limited (with *Lefty1* and *Fgfbp1*, being the only differentially expressed genes), suggesting that heterogeneity is a feature AVE cells acquire only during their characteristic migration and is directly related to the spatial positions the cells occupy within the embryos.

Finally, using the same sub-clustering approach, we showed that two transcriptionally distinct sub-clusters emerged within the 6.5-dpc exVE, differentially marked by *Nrg1* and *Efnb1*, while such distinctions were not detectable at 5.5 dpc (Figures 5F and 5G; Table S12). These exVE sub-clusters were spatially segregated to proximal (*Nrg1*+, *Efnb1*−) and distal (*Nrg1*− *Efnb1*+) regions of the 6.5-dpc exVE (Figure 5H) but showed overlap.

### Identification of signaling pathways important for AVE migration

**Figure 6:**
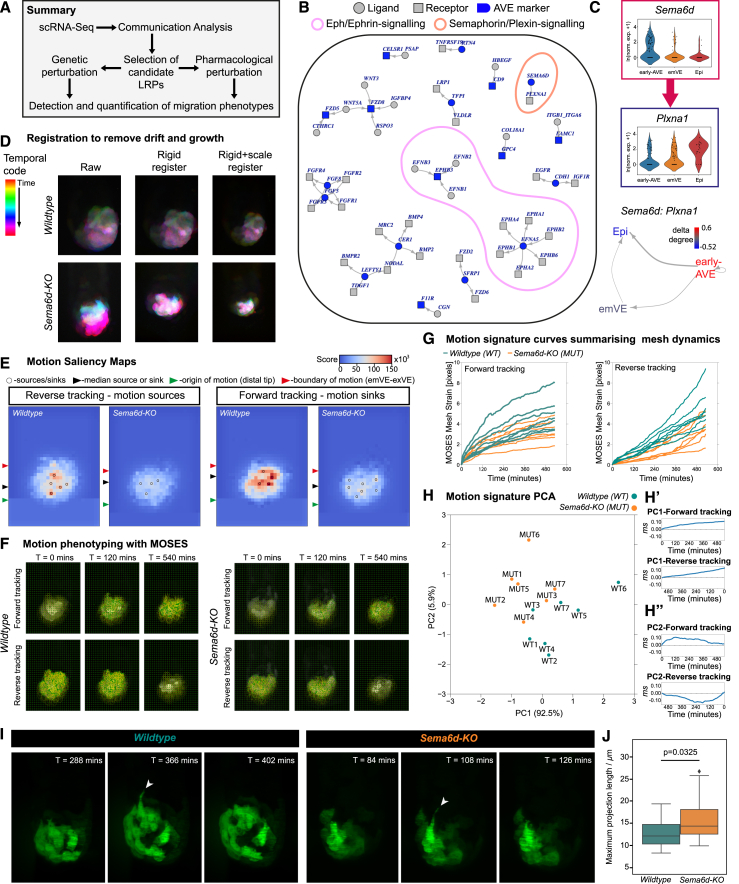
Semaphorin signaling is required for correct AVE migration (A) Schematic of computational and experimental strategy used to discover signaling pathways important for AVE migration. (B) Plot showing ligand-receptor pairs (LRPs) containing at least one high-in-AVE component (blue), at 5.5 and/or 6.25 dpc. Arrows indicate the direction of signaling. (C) Violin plots showing normalized log expression levels of *Sema6d* and *Plxna1* in AVE, emVE, and Epi clusters, at 5.5 dpc (top), and the intercellular communication pattern associated with SEMA6D:PLXNA1 (bottom). (D) Rigid body and scaling registration used to remove drift and growth from confocal time-lapse movies for phenotyping of AVE migration with MOSES analysis. (E) MOSES motion saliency maps computed from reverse and forward tracking (STAR Methods) to identify spatial location of motion sources (left) and sinks (right) from a wild-type and *Sema6d* homozygous mutant (*Sema6d-KO*) embryo. The position of the median source/sink relative to the origin and boundary of motion is marked. (F) Snapshots of the mesh connecting initially neighboring superpixels when MOSES is applied to track AVE migration (forward and reverse) in a wild-type and *Sema6d-KO* embryo. (G) Plots of motion signatures (mesh strain vs. time) extracted from tracking AVE migration in wild-type and *Sema6d-KO* embryos (*n* = 7 each), which summarize the extent of mesh deformation based on forward and reverse tracking. (H) Plot of the first two principal components (PC1 vs. PC2) after applying principal-component analysis to the values of the mesh strains extracted from the concatenated forward and reverse time-lapse tracking of AVE migration in wild-type (WT) and *Sema6d-KO* (MUT) embryos. In parentheses is the percentage of explained variance. (H′) and (H″) represent the loadings of PC1 and PC2, respectively, for the mesh strains obtained from the forward (top) and the reverse (bottom) tracking. The monotonic increase of PC1 suggests that it represents overall AVE movement. The non-monotonic behavior of PC2, with a stationary point at ∼120 min, indicates that it represents the rate at which AVE cells slow down. (I) Maximum intensity projections of high-resolution lattice light-sheet time-lapse frames from cultured wild-type and *Sema6d-KO* embryos, showing the emergence and retraction of basal projections by migrating Hhex-GFP-positive AVE cells (green). Arrowheads point to basal projections at their measured maximum lengths. (J) Plot showing difference (*p* = 0.03; nested ANOVA) in the maximum length of basal projections from wild-type (*n* = 9 embryos, *n* = 76 projections) and *Sema6d-KO* mutant embryos (*n* = 9, *n* = 66 projections). Data are represented as mean ± SEM. See also Videos S7 and S8.

Migration of the AVE is central to its function, but the processes controlling the initiation and directionality of migration are still unknown. By mining our scRNA-seq dataset, we sought to identify signaling interactions between the AVE and immediately surrounding tissues that might modulate the precise migratory behavior of the AVE (Figure 6A). We filtered down a list of 2,548 ligand-receptor pairs (LRPs^45^) to those most likely to mediate cell-cell communication (STAR Methods), encoded by genes from the high-in-AVE groups (Figure 1D). We then employed COMUNET^46^ for visualization and exploration of possible ligand-receptor interactions between the various cell types of the embryo at 5.5 and 6.25 dpc.

Supporting the validity of this approach, the analysis identified pathways previously known to play roles in AVE migration (fibroblast growth factor [FGF], Wnt, and transforming growth factor β [TGF-β] pathways). Additionally, this analysis identified putative interactions mediated by the bidirectional Ephrin/Eph signaling pathway (Figure 6B) that has not previously been implicated as playing a role in the pre-gastrulation embryo. A diversity of family members was found to be expressed with cell-type specificity, to the extent that even just a subset of genes belonging to this family was sufficient for separating all cell clusters previously identified using the entire transcriptome (Figures S5A–S5C; STAR Methods). *Efna5* and *Ephb3* showed relatively higher expression in the AVE (Figures S5D, S6A, and S6C) and were both found among the high-in-AVE genes (Tables S3 and S4). Potential communication patterns for *Efna5* and *Ephb3* at 5.5 dpc identified several plausible signaling interactions between the AVE and adjacent Epi or emVE cells (Figures S5E and S5F), raising the possibility that these short-range intercellular interactions might regulate AVE migration in some way.

Genetic KO of individual ephrins or receptors presumably does not affect AVE migration, as mutant embryos survive to and beyond gastrulation (e.g., Uziel et al.^47^). Given the high degree of redundancy within this large gene family, to test if this signaling pathway was collectively involved in AVE migration, we used a broad-spectrum pharmacological blockade of Ephrin/Eph signaling in 5.5-dpc embryos expressing the Hhex-GFP reporter. Control cultured embryos showed migration of AVE cells from the distal tip to the Epi-ExE boundary and then laterally across the embryo (*n* = 25/29). However, embryos cultured in the presence of the Ephrin/Eph signaling inhibitor NVP-BHG712^48^^,^^49^ showed a failure of AVE cell migration (*n* = 29/32) (Figure S6D). They also had more mitotic cells within the Epi and abnormal nuclear morphology in the VE, indicating that Ephrin/Eph signaling, in addition to regulating cell migration, plays a variety of important cell-type-specific roles in the 5.5-dpc embryo, consistent with the expression of these receptors and ligands across all cell types.

### Semaphorin signaling is required for normal AVE migration

Another LRP identified by our communication analysis that had not been investigated in the context of AVE migration was the bidirectionally signaling transmembrane proteins SEMA6D and PLXNA1 (Figures 6B and 6C). *Sema6d* is associated with the Gene Ontology (GO) term “cell migration” and was expressed at higher levels in the AVE, while its known receptor, *PlxnA1*, was mainly expressed in the surrounding emVE cells and Epi (Figures 6C, S6B, and S6E). Semaphorin-plexin interactions can be repulsive or attractive.^50^ *Sema6D* mutants have been previously reported to be post-natally viable,^51^ suggesting that they do not have a lethal AVE migration arrest. To test if SEMA6D has a more nuanced role in AVE migration, similar to proteins such as NAP1 that are required for AVE cells to migrate as a coherent flock,^23^ we cultured 5.5-dpc embryos expressing the Hhex-GFP transgene in the presence of an inhibitory antibody against SEMA6D (Figure S6F). Control embryos showed AVE migration as expected (*n* = 18/20), but most embryos cultured with the inhibitory antibody showed an arrest in migration (*n* = 21/26).

To investigate the nature of the phenotype more accurately, we engineered a *Sema6d* KO (*Sema6d*-*KO*) mouse line containing the *Hhex-GFP* AVE reporter. We monitored AVE migration in *Sema6d* homozygous KO embryos (MUT; *n* = 7), alongside their wild-type littermates (WT; *n* = 7), using time-lapse confocal microscopy. Visual inspection of AVE migration in mutants suggested that migration might be occurring slower than normal (Video S7). To quantitatively determine if there were differences in the migration dynamics of AVE cells in mutants without a priori assumptions relating to aspects of migration that might be defective, we used motion sensing superpixel (MOSES) analysis^52^^,^^53^ (Figures 6D–6F). MOSES extracts cellular motion by equipartitioning the image into non-overlapping regions of interest, or superpixels, and tracking them over time. By joining together spatially neighboring superpixel tracks, MOSES builds a dynamic mesh to quantify motion phenotypes (Figures 6E–6H; STAR Methods). We applied MOSES to our time-lapse volumes, tracking Hhex-GFP AVE cells backward in time (reverse tracking) to capture motion sources (the origin of migration) and forward in time (forward tracking) to capture motion sinks at the end of proximal migration.^52^ The computed motion saliency maps showed that as expected, WT embryos exhibit distinct motion sources closer to the distal tip, and motion sinks near the Epi-ExE boundary, which correspond, respectively, to the start- and endpoint of AVE migration (Figure 6E). In contrast, *Sema6d* mutants showed less distinctly identifiable motion sources and sinks, pointing to a profound defect in ordered migratory behavior of AVE cells.

Video S7. Comparison of AVE migration in a wild-type and a homozygous *Sema6d*-knockout embryo, related to Figure 6 Confocal time-lapse imaging of *Hhex-GFP-*expressing AVE cells in a wild-type (left) and *Sema6d* homozygous knockout embryo (right), isolated at 5.5 dpc and cultured for 9 h. Images were acquired every 6 min, and videos show maximum intensity projection of optical slices. The embryos are orientated with the anterior on the left and the distal tip at the bottom.

To quantitatively separate mutant and WT embryos based on their motion signatures and determine the specific aspect of AVE migration that was defective, we computed the MOSES mesh strain for every time point in each of our confocal time-lapse volumes. This allowed us to plot a mesh strain curve for each embryo, which captures the mean mesh distortion relative to the initial relaxed mesh and reflects the extent of relative movement between individual AVE cells (Figures 6F and 6G; STAR Methods). Even within any one embryo, this can differ for forward and reverse tracking, because the reference mesh (taken from the starting time point of the time series) will be different. Comparison of mesh curves of WT and mutant embryos showed a clear separation based on genotype (Figure 6G). A principal-component analysis of the motion signatures (the MOSES mesh strain at each time point from both forward and reverse tracking, equivalent to 180 dimensions) for each embryo indicated a clear separation in migration phenotype between WT and mutant embryos along two principal components (PC1 and PC2; Figure 6H). The loadings of PC1 and PC2 (Figures 6H′ and 6H″) show that PC1, which accounts for over 90% of the difference between WT and mutant embryos, corresponds to the generalized movement associated with AVE migration, based on its monotonic linear increase (Figure 6H′). PC2 accounts for ∼6% of the difference and corresponds to the rate at which AVE cells slow down toward the end of their migration, based on the distinct inflection in the mesh strain curve at approximately 120 min into AVE migration (Figure 6H″). The migratory phenotype of *Sema6d* mutant AVE cells showed a reduction in PC1 and an increase in PC2. Altogether, our motion phenotyping shows that in WT embryos, AVE cells “surge” forward proximally and then decelerate as they approach the Epi-ExE boundary. In contrast, AVE cells in *Sema6d* mutants showed a marked reduction in the speed with which they advanced toward the boundary.

To understand the cellular phenotype associated with the migratory defect in *Sema6d* mutants, we used lattice light-sheet microscopy to time-lapse image AVE migration at higher resolution. This allowed us to visualize basal projections produced by the most anterior AVE cells (i.e., between Hhex-GFP-labeled AVE and -unlabeled emVE/exVE cells) (Figure 6I; Video S8). Quantification of the length of these projections showed that they were significantly longer (*p* = 0.0325; nested ANOVA), extending across the Epi-ExE boundary in *Sema6D*-*KO* embryos (*n* = 12), compared with WT littermates (*n* = 10; Figures 6I and 6J). This was indicative of a more exploratory nature of migrating AVE cells in the absence of SEMA6D, which might cause their stalled migratory progression.

Video S8. Lattice light-sheet timelapse imaging of basal projections during AVE migration, related to Figure 6 Lattice light-sheet time-lapse imaging of migrating *Hhex-GFP-*expressing AVE cells in a wild-type embryo isolated at 5.5 dpc and cultured for 9 h. The embryos were illuminated with a 100 μm × 1, 800 μm Sinc3 beam and imaged at 5-min time resolution. The video is a maximum intensity projection of 643 optical sections after deskewing and coverglass correction over the first 7 h of culture. The embryo is orientated with the anterior on the left and the distal tip at the bottom.

## Discussion

Collective directional migration of the AVE is a precise and highly coordinated process.^7^^,^^54^ It plays a pivotal role in specifying the first definitive axis of the body, the AP axis, upon which all further development is predicated. By coupling complementary experimental techniques, we showed that this dynamic nature of the AVE, overtly observed as its migratory behavior and transient function, emerges from an elaborate series of continually changing molecular heterogeneities.

AVE cells are known to have basal projection in the direction of migration,^7^^,^^14^^,^^22^ but the role of such projections in AVE migration remains unclear. We identify a molecular signaling interaction that regulates the length of these basal projections, and we characterize the way in which this alteration in the projections leads to impaired AVE migration. This reinforces that it is not only apical junctional events^20^ but also projections from the basal domain that are important in the migration of AVE cells in an epithelial context.

Semaphorins and their receptors are mainly membrane-tethered molecules. They were first identified as axon guidance cues but have since been shown to regulate the migration of other cell types by direct intercellular signaling.^55^^,^^56^^,^^57^^,^^58^ In the follicular epithelium of *Drosophila* egg chambers, transmembrane semaphorins can be planar polarized and enriched at the leading edge of the basal surface of cells, from where they coordinate unidirectional collective migration through communication with the cells ahead, expressing the corresponding plexin receptor.^59^ During mammalian heart development, semaphorins act as the regulatory target of TGF-β signaling, activating members of the Rho family of small GTPases.^60^ Rho-GTPase signaling has also been shown to be involved in regulating basal projections during AVE migration,^22^ opening up the possibility that semaphorins could act upstream of GTPases to mediate signaling in this context. Furthermore, during chick cardiogenesis, SEMA6D can either promote or inhibit endocardial cell migration, through co-receptor-specific attractive or repulsive interactions.^50^^,^^58^

From our data, we hypothesize that SEMA6D in migrating AVE cells acts as a repulsive cue between the AVE and surrounding emVE and exVE cells, both of which express the PLXNA1 receptor. In the absence of signaling through SEMA6D, presumably repulsive interactions at the boundary are dampened, resulting in the observed increase in basal projection length, curtailing their unidirectional advancement.

Similarly, Eph-Ephrin interactions can regulate the directional nature of cell movements through coordinating contact inhibition.^61^^,^^62^^,^^63^ They are also necessary for boundary formation between cells^64^^,^^65^ and have been suggested to play a role in germ layer separation in the mouse gastrula.^66^ Differentially expressed *Eph* and *Ephrins* in the cell types of the early embryo, in addition to facilitating AVE migration, could be the molecular basis for the Epi-ExE/emVE-exVE boundary that marks the proximal extent of AVE migration. In *Xenopus*, Ephrin-Eph-based repulsion is utilized to establish sharp tissue boundaries with high interfacial tension.^67^ During AVE migration, tensional boundaries that match migration boundaries have been recently observed.^68^ These boundaries overlap with the Ephrin-Eph boundaries we report in this study, making them promising candidates as mediators of not just cell-type segregation but also cell behaviors within tissues.

The boundary to which the AVE migrates fixes an AP axis orthogonal to the existing PD axis of the egg cylinder, but why the AP axis does not then rotate or drift distally with the lateral displacement of the AVE cells is unknown. We show that as AVE cells move laterally, they downregulate their AVE-specific transcriptional program and revert to a transcriptional state similar to that of the cells from which they originate. While AVE cells at the anterior possess the most “mature” AVE transcriptional profile, the gradual attenuation of anteriorizing gene expression with lateral displacement would be necessary to restrict Wnt and Nodal repression to the anterior. Such tight coupling of transcriptional state and position is presumably required because of the relatively small egg cylinder, within which patterning must be achieved through diffusible morphogens in a precise and reproducible manner. This also indicates that the AVE at 5.5 and 6.5 dpc, albeit composed of broadly the same cell types, captures different transcriptional states and exhibit different levels of “maturity.” The transcriptional state observed among the 6.5-dpc AVE (the most transcriptionally distinct and mature), is thus a transient one, acquired by migrating VE cells, and is directly related to the position the cells occupy in the embryo.

Finally, to add to the molecular description of AVE migration, we performed the first phosphoproteomic characterization of the peri-gastrulation mouse embryo. Leveraging the phosphoproteomic against the scRNA-seq data helped us bridge the transcriptome and the functional proteome. We identified several proteins enriched or differentially phosphorylated within the small AVE population, such as KRT8, DBN1, MARCKS, and MARCKSL1, known to control the morphology and motility of a wide range of cell types through modulating cytoskeletal dynamics.^33^^,^^35^^,^^69^^,^^70^ Sub-cortical F-actin is enriched in a ring delineating the apical junctions of emVE cells,^20^ and regulators of F-actin branching^14^^,^^23^ are essential for normal AVE migration. Overall, the close coordination between intercellular communication and cell-autonomous modulation of cytoskeletal dynamics could ensure precision and reproducibility to AVE migration.

### Limitations of the study

The depth of our proteomics and phosphoproteomics study is limited due to the small size of early embryos restricting the amount of material that can be practically collected. As a result, fewer differentially expressed proteins and phosphoproteins were identified in comparison with the transcriptomics. Although we analyzed the proteomics results in light of transcriptomic data that were obtained at a single-cell resolution, it is likely that proteins expressed at low levels and posttranslational modifications specific to sub-populations of cells might have been missed. Additionally, one of the major signaling pathways identified using the communication analysis was involving Ephrins and Eph receptors. Due to the number of family members from this pathway implicated in possible communication networks involving the AVE, we utilized pharmacological, rather than genetic, blockage of Ephrin/Eph signaling. This possibly resulted in off-target effects on the embryo.

## STAR★Methods

### Key resources table

**Table:** 

REAGENT or RESOURCE	SOURCE	IDENTIFIER
**Antibodies**		

Rabbit anti-CK19	Proteintech	10712-1-AP; RRID: AB_2133325
Rabbit anti-Drebrin	Proteintech	10260-1-AP; RRID: AB_2230301
Rabbit anti-MARCKS	Proteintech	10004-2-Ig; RRID: AB_2140311
Rabbit anti-MARCKSL1	Proteintech	10002-2-AP: RRID: AB_513892
Rabbit anti-CK8	Proteintech	10384-1-AP; RRID: AB_10638912
Rabbit anti-phospho(Ser23)CK8 EP1629Y	Abcam	ab76584; RRID: AB_2049847
Goat anti-PlexinA1	R&D Systems	AF4309: RRID: AB_10645644
Mouse anti-Sema6D	SantaCruz	sc393258; RRID: AB_3099689
Rabbit anti-OCT4	Abcam	ab19657; RRID: AB_445175
AF-555 Donkey anti-rabbit IgG	Invitrogen	A31572; RRID: AB_162543
AF-633 Donkey anti-goat IgG	Invitrogen	A21082; RRID: AB_2535739

**Chemicals, peptides, and recombinant proteins**		

RNaseZap™	Invitrogen	AM9760
M2 medium	Sigma-Aldrich	M7167
Claret FR Fluorescent Cell Linker Kit	Sigma-Aldrich	MINCLARET-1KT
TrypLE™ dissociation reagent	Invitrogen	12563011
Heat-inactivated FBS	Thermo-Fisher	10500
Donkey serum	Sigma-Aldrich	D9663
BSA	Sigma-Aldrich	A7906
DAPI	Thermo Fisher	D1306
Proteinase-K	Thermo-Fisher	EO0491
Paraformaldehyde	SantaCruz	CAS 30525-89-4
Glycerol	Fisher Scientific	G/0650/17
VectaShield	Vector Labs	H-1200
Phalloidin-Atto 550	Sigma-Aldrich	19083
Phalloidin-Atto 647N	Sigma-Aldrich	65906
HCR probe sets, amplifiers, and buffers	Molecular Instruments	HCR RNA-FISH Bundles
HBSS	Sigma-Aldrich	55037C
CMRL-1066 medium	Pan BioTech	P04-84600
KnockOut serum replacement	Gibco	10828-010
NVP-BHG712	Selleck Chemicals	A8683
Triton X-100	Sigma-Aldrich	T8787
Tween-20	Sigma-Aldrich	P1379

**Deposited data**		

On ArrayExpress: Single-cell RNA-Seq of VE-enriched cell populations from E5.5 and E6.25 wild type mouse embryos	This paper	ArrayExpress: E-MTAB-9645
Code repository	This paper	Zenodo: 10829359
10x scRNA-Seq data	Nowotschin et al.^17^	https://explore.data.humancellatlas.org/projects/4e6f083b-5b9a-4393-9890-2a83da8188f1

**Experimental models: Organisms/strains**		

Mouse: CD1	Charles River, England	Strain code: 022
Mouse: C57BL/6	In house; University of Oxford, Biomedical Services	N/A
Mouse: Hhex-GFP	Rodriguez et al.^28^	N/A
Mouse: Sema6D-KO	This paper: *see*STAR Methods	N/A

**Oligonucleotides**		

*Sema6d* sgRNA [CUGCGAUUCGUUCGGU GAA+tracrRNA]	Sigma-Aldrich	MMPD0000123097
*Sema6d* F primer [5’-CAGCAGCCCAGACA TAGAGA-3’]	Invitrogen	N/A
*Sema6d* R primer [5’-TGCAAGCACCACAA GAGAAA-3’]	Invitrogen	N/A

**Software and algorithms**		

Volocity, Improvisions (version: 6.3)	N/A	https://www.volocity4d.com/
Fiji ImageJ2 (version: 2.14.0/1.54f)	N/A	https://imagej.net/software/fiji/
GraphPad Prism for Windows (version: 9.5.1)	GraphPad Software Inc.	https://www.graphpad.com/features
Salmon (version: 0.13.1)	Patro et al.^71^	https://github.com/COMBINE-lab/salmon
STAR (version: 2.7.0f_0328)	Dobin et al.^72^	https://github.com/alexdobin/STAR
Velocyto (version: 0.17.17)	La Manno et al.^43^	https://velocyto.org/velocyto.py/
R (version: 3.5.2)	N/A	https://posit.co/download/rstudio-desktop/
Python (version: 3.9.5)	N/A	https://www.python.org/downloads/release/python-395/

**Other**		

35 mm glass bottom dishes	MatTek Life Sciences	P35-1.5-14-C
Nunc™ Lab-Tek™ II chambered coverglass slides	Thermo Fisher	155409
8-Well μ-Slide #1.5	Ibidi	IB-80807

### Resource availability

#### Lead contact

Further information and requests for resources and reagents should be directed to and will be fulfilled by the lead contact, Shankar Srinivas (shankar.srinivas@dpag.ox.ac.uk).

#### Materials availability

Cryopreserved sperm from the *Sema6d* knockout mouse line generated for this study is available upon request from the lead contact. No other new unique reagents were generated for this study.

#### Data and code availability

• The data have been deposited at ArrayExpress and are publicly available under accession number E-MTAB-9645 as of the date of publication.
• All original code can be publicly accessed at https://github.com/ScialdoneLab/scAVE as of the date of publication.
• Any additional information required to reanalyse the data reported in this paper is available from the lead contact upon request.

### Experimental model and study participant details

#### Mouse strains and husbandry

All animal experimentation procedures were performed in full accordance with the UK Animals (Scientific Procedures) Act 1986, approved by Oxford University’s Biological Services Ethical Review Process and were performed under UK Home Office project licenses PPL 30/3420 and PCB8EF1B4. Mice were maintained on a 12h light, 12h dark cycle, with *ad libitum* access to food and water, and under animal husbandry and housing conditions as approved by the UK Home Office. Noon on the day of finding a vaginal plug was designated 0.5 days post coitum (dpc). For the various experiments detailed below, C57BL/6J (in house) or CD1 (Charles River, England) females crossed to C57BL/6J or homozygous *Hhex-GFP* transgenic studs.^28^

The *Sema6d* knockout line was generated on a C57BL/6J background homozygous for the *Hhex-GFP* reporter allele using a CRISPR-Cas9 approach as previously described^73^ with the following modifications. 1-cell embryos were microinjected with 100ng/μl Cas9-mSA mRNA and 50ng/μl sgRNA [CUGCGAUUCGUUCGGUGAA+tracrRNA; MMPD0000123097, Sigma-Aldrich] targeting exon-2 of the *Sema6d* gene. Injected embryos were transferred into pseudopregnant foster mothers. The pups once born were screened and mutant alleles were identified by Sanger sequencing the *Sema6d* locus. The mice derived from the injected/fostered embryos were genetically mosaic with multiple *Sema6d* alleles. Alleles were segregated by crossing F1 to *Hhex-GFP* homozygous/*Sema6d* wildtype mice. An allele with a 14bp deletion [ΔCCCTTCAGGCAACG], was selected to establish the line. PCR using F [5’-CAGCAGCCCAGACATAGAGA-3’] and R [5’-TGCAAGCACCACAAGAGAAA-3’] primers were subsequently used to genotype the wildtype and mutant alleles amplifying products that were 386bp and 372bp respectively, separated by electrophoresis on a 2.5% (w/v) agarose gel.

### Method details

#### Embryo collection

Embryos were collected at the appropriate stages between 5.25-6.5 days post coitum (dpc). Since there is considerable natural variation in the extent of development even within litters at the stages studied (see Figures S3A and S3B and Table 3.1 in Lawson and Wilson^4^), and AVE migration happens over a period of several hours, for HCR and immunofluorescence experiments we staged embryos more precisely, based on the extent of AVE migration. The columnar morphology of the AVE cells (or where relevant, Hhex-GFP expression) was used as the metric to determine the position of the AVE relative to the distal tip and the epiblast–ExE boundary. At 5.5 dpc, embryos where the AVE was at the distal tip were staged as pre-migration and those in which the AVE was at a position between the distal tip and the epiblast–ExE boundary, were staged as mid-migration. 6.25 dpc embryos where the AVE had reached the epiblast–ExE boundary, were staged as post-migration. Both the columnar morphology of cells and the epiblast–ExE boundary were visualised under transmitted light. The dissections were done according to a standard post-implantation dissection protocol as previously described.^74^ All dissecting instruments were thoroughly cleaned with RNase*Zap*™ (Invitrogen, AM9760) and 70% ethanol, and embryos being collected for HCR were kept in ice-cold M2 medium (Sigma-Aldrich, M7167) throughout.

#### Single cell isolation, cDNA library preparation and sequencing

Embryos were collected at 5.5 (*n* = 40) and 6.25 dpc (*n* = 11) from C57BL/6J females crossed to C57BL/6J studs. We reasoned that collecting at these two broad stages would allow us to capture AVE cells from before migration till well after the end of their proximal migration. Embryos show considerable natural variation in the extent of development even within a single litter, so collecting from multiple litters at these two *days post coitum*, helped ensure that we covered not only the "start" and the "end" but also intermediate points of AVE migration. Furthermore, even within a single embryo, cells are found in a *range* of transcriptional states, that cover multiple developmental stages (as detailed in Figure 1G of Mittnenzweig et al.^75^).

To enrich for VE cells in the single-cell collection, fluorescent membrane labelling of the VE was achieved using the CellVue Claret Far Red Fluorescent Cell Linker Kit (Sigma-Aldrich, MINICLARET-1KT). Briefly, embryos were incubated in 0.1% (v/v) Claret Far Red dye in Diluent-C for 5 minutes at room temperature (RT), which was sufficient to label primarily the outer cell layers of the embryo. The labelling reaction was stopped with an equal volume of 1% BSA, and then rinsed with M2 medium. Up to 4 stained embryos were placed in 100μl of TrypLE™ dissociation reagent (Invitrogen, 12563011) for 3.5 minutes and 4.5 minutes for 5.5 and 6.25 dpc embryos respectively, at 37°C. The embryos were then mouth-pipetted up and down, for gentle mechanical dissociation using a glass capillary 10-15% larger than the size of the embryos. The dissociated cells were pooled and transferred to a 1.5 mL microcentrifuge tube and the TrypLE was neutralised with an equal volume of heat-inactivated FBS (Thermo Fisher, 10500) followed by centrifugation at 1000x *g* for 3 minutes at 4°C. The cells were resuspended in 100μl of ice-cold HBSS (Sigma-Aldrich, 55037C) with 1% FBS. DAPI (0.1 *μ*g/mL; Vector Labs, H-1200) was added as a live-dead indicator. Claret-labelled, live, VE cells were collected using SH800 Cell Sorter (Sony Biotechnology) directly into plates containing lysis buffer at 4°C.

Total mRNA from the cells were extracted and amplified using the SMARTSeq2 protocol^76^ with the additional inclusion of ERCC spike-in control at 1/10^7^ concentration. Multiplexed sequencing libraries were generated from cDNA using the Illumina Nextera XT protocol. 125 bp paired-end sequencing was performed on an Illumina HiSeq 2500 instrument (V4 Chemistry).

#### Transcript quantification, quality control and normalization

We performed transcript quantification in the scRNA-seq datasets from stages 5.5 and 6.25 dpc, employing Salmon v0.13.1,^71^ in the quasi-mapping-based mode. First, we created a transcriptome index from the mouse reference (version GRCm38.p6) and ERCC spike-in sequences. Then, we used the “quant” function to quantify the transcripts, correcting for the sequence-specific biases (“--seqBias” flag) and the fragment-level GC biases (“--gcBias” flag). Finally, we aggregated the transcript level abundances to gene level counts. The obtained raw count matrices include 384 samples at each stage (5.5 and 6.25 dpc).

Afterwards, we performed a quality control to eliminate low quality cells from downstream analyses. We selected good quality cells according to the following criteria (same for both 5.5 and 6.25 dpc):

Number of genes with more than 10 reads per million (rpm) larger than 3,000:

- Log_10_ of the total number of reads larger than 4;
- Fraction of mapped reads larger than 0.5;
- Fraction of reads mapped to mitochondrial genes smaller than 0.1;
- Fraction of reads mapped to ERCC spike-ins smaller than 0.3.

With these criteria, we obtained 255 good quality cells at 5.5 dpc and 238 cells at 6.25 dpc.

We normalized the raw count matrices separately at 5.5 and 6.25 dpc using the R package “scran” v1.10.2,^77^ with default parameters, and we log-transformed the data (adding a pseudocount of 1 in order to avoid infinities) using the natural logarithm function “scanpy.pp.log1p” in Scanpy v1.425.^78^

#### Cell clustering and assignment of cluster identities

We performed hierarchical clustering of the cells from each stage separately, using an information theoretic criterion to guide the choice of the number of clusters, as described below. First, we computed the highly variable genes (HVGs) employing the Scanpy function “scanpy.pp.highly_variable_genes”, with default parameters except for “max_mean” (set to 10), and retained the top 3,000 genes at both 5.5 and 6.25 dpc stages.

Then, we computed the distance matrix between cells as $\sqrt{\left(1-\rho \right)/2}$, where ρ is the Spearman’s correlation coefficient between cells. Hierarchical clustering was carried out on this distance matrix (function “hclust” in R, with average agglomeration method) and we cut the dendrogram with the dynamic hybrid cut method (“cutreeDynamic” function in the R package “dynamicTreeCut” v1.63.1, with the hybrid method and a minimum cluster size of 10 cells;^79^). This method depends on the parameter “deepsplit”, which ranges between 0 and 4 and determines the number of clusters.

To estimate the number of clusters, for each value of “deepsplit”, we computed the average Variation of Information^80^ between the clustering obtained using the top 3,000 HVGs and 50 subsamples in which only half of the genes is randomly kept. Similar to the elbow method, we chose the largest “deepsplit” value before the average Variation of Information has a ‘kick’ towards high values (see for instance Figure S1C). The Variation of Information is computed using the function “vi.dist” in R package “mcclust” v1.0 (Fritsch and Ickstadt, 2009). This procedure gave 4 clusters at 5.5 dpc and 3 clusters at 6.25 dpc.

At 5.5 dpc, two clusters corresponded to Visceral Endoderm (VE) cells, which, based on their marker genes, could be identified as the VE portions covering the extraembryonic ectoderm (exVE) and the epiblast. We further divided the latter into two clusters by recomputing the HVGs and by employing the “Partitioning Around Medoids” (pam) function (R package “cluster” v2.1.0). The function was run on the Spearman’s correlation distance matrix, computed as described above. This allowed us to distinguish an AVE cluster from the rest of the VE covering the epiblast (emVE).

At 6.25 dpc, while one cluster shows a clear AVE signature, the other two include multiple cell populations, as can be seen from the expression of marker genes. In particular, the first includes exVE and emVE together, which we separated by recomputing the HVGs and by applying the “pam” function as described above. The second cluster mostly includes epiblast cells, while a few cells express extraembryonic ectoderm (ExE) markers. We identified the ExE cells using an outlier detection algorithm based on the distance from the k-nearest neighbours for each cell python package “PyOD”: https://pyod.readthedocs.io/en/latest/index.html,^81^ function “KNN” in “pyod.models.knn”.

Overall, we identified 5 clusters each in 5.5 and 6.25 dpc embryos: one corresponding to epiblast cells (Epi), another including cells from the extra-embryonic ectoderm (ExE) and three clusters of visceral endoderm cells (emVE, exVE and AVE). Three cells at each stage were unassigned in the clustering analysis; we eliminated them for downstream analyses, ending up with 252 cells at 5.5 dpc and 235 at 6.25 dpc.

We computed markers for the clusters relying on the Scanpy function “scanpy.tl.rank_genes_groups”. For each pair of clusters, we tested the differential expression of genes using the Wilcoxon test, with Benjamini-Hochberg correction for multiple testing. We selected genes with log_2_ fold change larger than 1 and adjusted *p*-value smaller than 0.1. For each cluster, we ranked the genes based on their average -log_10_ of the adjusted *p*-values across all pairwise comparisons. The heatmaps (Figure S1) were generated considering the top five markers per cluster. Note that some genes can be markers of more than one cluster. The top 50 markers per cluster at the two stages are listed in Tables S1 and S2. The UMAPs at 5.5 and 6.25 dpc were generated using the Python package “umap” v1.3.9,^82^ with 30 nearest neighbours using the same distance matrix that was used for clustering.

For the computation of the relative distances between VE cluster centroids (Figure S1D), we identified the centroids using the “NearestCentroid” function in the Python package “scikits-learn” v0.21.3. Then, we computed the Spearman’s correlation distance between them, as described above. The PAGA graphs for the VE clusters at the two stages (Figure S1D), were computed with the Scanpy function “scanpy.tl.paga”^25^.

#### Diffusion pseudotime analysis of AVE and emVE cells

We selected only AVE and emVE cells at each stage and we identified the top 3,000 HVGs as described above. Using these genes, we performed a Principal Component Analysis (PCA) (Scanpy function “scanpy.tl.pca”, with “arpack” as SVD solver) and we built a k-nearest neighbour (knn) graph of the cells (Scanpy function “scanpy.pp.neighbors” with k=15) based on the Spearman’s correlation distance calculated on the first 10 Principal Components.

Starting from this knn graph, we computed a diffusion map (Scanpy function “scanpy.tl.diffmap”) and a pseudotime coordinate (Scanpy function “scanpy.tl.dpt”), choosing as the root cell the one with minimum and maximum value of the second Diffusion Component (DC) at 5.5 and 6.25 dpc, respectively.

To find genes differentially expressed in pseudotime, first we filtered out genes detected in fewer than 10 cells. Then, we employed a Generalized Additive Model (R function “gam” from “GAM” package v1.16.1) to fit the expression in pseudotime of each gene and we computed a p-value using the ANOVA test for parametric effects provided by the “gam” function. After FDR correction, we obtained 952 differentially expressed genes at 5.5 dpc and 915 at 6.25 dpc (FDR < 0.01).

We classified the differentially expressed genes based on their trend in pseudotime. To do so, we clustered genes with the same approach used for cell clustering, but this time we fixed a minimum cluster size of 50 and the random samples for the computation of the average Variation of Information were obtained by randomly sampling 70% of cells from the dataset 50 times. We identified two groups of differentially expressed genes at each stage (with deepsplit = 2 and 1 at 5.5 and 6.25 dpc, respectively), one of genes with decreasing expression in pseudotime (“high-in-AVE genes”) and the other with increasing expression in pseudotime (“low-in-AVE genes”).

The lists of the differentially expressed genes with gene group assignment at each stage are reported in Tables S3 and S4.

#### Isoform analysis of scRNA-seq data

We used Salmon to obtain the isoform-level count matrix with ENSEMBL reference (version GRCm38.p6) for annotation and we normalized counts using transcripts per million (TPM) normalization. We removed genes with more than 80% of the counts mapped to a single isoform. Next, we compared transcript levels between each pair of clusters at 5.5 and 6.25 dpc. We built a contingency table for each gene with the average normalized expression levels of each isoform in the pair of clusters being compared. Finally, following Tyser et al.^83^ and Froussios et al.,^84^ we used a chi-squared test to find differentially expressed isoforms between the two clusters for a given gene. We report the genes with differential isoform expression between early/late-AVE and emVE and that were not high-in-AVE or low-in-AVE in the diffusion pseudotime analysis in Table S13.

#### Proteomics and phosphoproteomics analysis

A total of 104 embryos were dissected at 6.25–6.5 dpc (taking into account variations within the litters when collecting such large number of embryos) as previously described.^74^ Using fine tungsten needles, the embryos were carefully bisected along the epiblast–ExE boundary and the embryonic and abembryonic halves generated were pooled separately. The embryonic half (EPI half) included the epiblast and the visceral endoderm surrounding it; the abembryonic half (ExE half) had the extra-embryonic ectoderm and the associated visceral endoderm cells (see Figure S1B). Sample preparation for proteomics was carried out as previously described.^85^ Cells from both pools were harvested, lysed, and treated with phosphatase inhibitors. Each pool was further divided into four aliquots that were processed individually as technical replicates. Further treatment on resultant peptide solutions included enrichment of phosphopeptides via Immobilized Metal Ion Affinity Chromatography IMAC.^86^ We analysed the phosphoproteomes using liquid choromatography-tandem MS (LC-MS/MS) as previously described.^87^ Briefly, phosphopeptide pellets were resuspended in 20μl of 0.1% TFA, and 4μl was loaded into an LC-MS/MS system, which consist of a nanoflow ultrahigh pressure liquid chromatography (UPLC, nanoAccuity Waters) coupled online to an Orbitrap XL mass spectrometer (ThermoFisher Scientific). Each sample was run three times and the data for each sample was averaged over the three runs, to generate chromatogram data.

In the proteomics and phosphoproteomics experiments, 1,690 and 1,770 peptides were quantified, respectively (see Tables S14 and S15 for the raw counts). We aggregated the peptide data to the protein level by summing the counts of peptides corresponding to the same protein. The data was normalized using the “normalyzer” function from the R package “NormalyzerDE” v1.0.0,^88^ which compares several quality metrics for different normalization methods. Following the analysis presented in Chawade et al.,^89^ we chose the Loess method. For each sample, we averaged the expression levels of the proteins over the three runs.

We performed a differential expression test between the embryonic and abembryonic halves for all the proteins in each dataset, using the function “normalyzerDE” from the same package mentioned above. For each protein, the function returns the log_2_ fold change between the embryonic and abembryonic halves and the adjusted *p*-value. We ran a functional enrichment analysis using the R package “gprofiler2”^90^ on the proteins that are significantly upregulated in the embryonic or abembryonic halves from the proteomics and the phosphoproteomics datasets. The results are reported in Tables S16 and S17. In Figure S2G we show the Manhattan plots with the significantly enriched terms of interest highlighted, obtained using the functions “gostplot” and “publish_gostplot” from “gprofiler2”.

We compared the proteomics and phosphoproteomics results by plotting the log_2_ fold changes in a scatter plot (Figure 2A), for the proteins quantified in both datasets. Proteins are marked as differentially expressed if the log_2_ fold change is larger than 1 (in absolute value) and the adjusted *p*-value is smaller than 0.1. Proteins are labelled as “high-in-AVE” if the corresponding gene was found in the “high-in-AVE genes” group from the diffusion pseudotime analysis of the scRNA-seq data from AVE and emVE cells at 6.25 dpc (see above). In the scatter plot, we also distinguish proteins with only one peptide in the dataset from those with multiple peptides.

We performed a kinase-substrate network analysis on the proteins that are differentially phosphorylated between the embryonic and abembryonic halves in the phosphoproteomics dataset. We considered known kinase-substrate interactions in mice from the PhosphoSitePlus database^91^ and we selected interactions involving substrates that are differentially phosphorylated in our dataset, checking also for the correspondence of the phosphorylation sites. We obtain two bipartite kinase-substrate networks for the embryonic and abembryonic halves, shown in Figure 2D.

To compare the proteomics and phosphoproteomics data with the scRNA-seq data, the cells at 6.25 dpc were split into two groups, based on whether they belong to the “embryonic half” (Epi, emVE and AVE cells) or the “abembryonic half” (ExE and exVE). After removing the genes detected in fewer than 10 cells, we identified the differentially expressed genes between these two groups of cells with the R package “DESeq2.”^92^

We represented the results in two scatter plots, showing the estimated log_2_ fold change in the scRNA-seq data on the *x*-axis and the log_2_ fold change in the proteomics (Figure S2D) or phosphoproteomics data (Figure S2E) on the *y*-axis. Genes/proteins are marked as differentially expressed if the log_2_ fold change is larger than 1 (in absolute value) and the adjusted *p*-value is smaller than 0.1. As before, genes/proteins are labelled as “high-in-AVE” if the corresponding gene was found in the ‘high-in-AVE’ genes group from the diffusion pseudotime analysis of the scRNA-seq data from AVE and emVE cells at 6.25 dpc (see above).

#### RNA velocity of AVE and emVE cells

Firstly, we selected AVE and emVE cells from the 5.5 and 6.25 dpc datasets. Then, we integrated the data from the two stages using the “mnn_correct” function in the “mnnpy” Python package (method previously introduced^93^, the Python implementation is available at https://github.com/chriscainx/mnnpy), using the intersection of the top 3,000 HVGs from the two stages. We scaled the data to zero mean and unit variance (Scanpy function “scanpy.pp.scale”, with max_value=10), and we computed a diffusion map with the same procedure as above (with 20 principal components and k=15).

To perform the analysis of RNA velocity, we generated the raw count matrices of spliced and unspliced counts at 5.5 and 6.25 dpc, using “STAR” v2.7.0f_0328^72^ and “velocyto” v0.17.17,^43^ in “run-smartseq2” mode. We merged the matrices and performed the downstream analysis using the Python package “scvelo” v0.2.2^44^ as described below. We filtered out genes expressed in fewer than 10 cells, then we used the scvelo function “scvelo.pp.filter_and_normalize”, with parameters “min_counts”=20 and “min_counts_u”=10, to filter genes on the basis on their number of spliced and unspliced counts, before normalizing the raw count matrix and selecting the top 3,000 HVGs.

We used the function “scvelo.pp.moments” to compute neighbouring cells and spliced and unspliced moments. After this, the inference of the genes’ splicing dynamics and the computation of the RNA velocities were computed from the dynamical model (using the functions “scvelo.tl.recover_dynamics”; “scvelo.tl.velocity” with “dynamical” mode; “scvelo.tl.velocity_graph”). Finally, we projected the velocities onto the first two DCs of the previously computed diffusion map (using the function “scvelo.tl.velocity_embedding”) and we computed the PAGA velocity graph using the function “scvelo.tl.paga” with default parameters.

#### Sub-clustering of Visceral Endoderm cell populations

We performed a sub-clustering analysis of the AVE and emVE cells in a previously published scRNA-seq dataset^17^ obtained through the 10x protocol. We downloaded the raw count matrix as an AnnData object and the metadata from https://explore.data.humancellatlas.org/projects/4e6f083b-5b9a-4393-9890-2a83da8188f1. We selected only VE cells from 5.5 and 6.5 dpc embryos (annotated as E5.5 and E6.5 in their study), which were collected in three and two batches, respectively. We normalized the data separately for each stage and batch using the R package “scran”, and we log-transformed the matrices as described above. At each stage, we integrated the data from the batches using the “mnn_correct” function from the “mnnpy” package (see above).

In Nowotschin et al.,^17^ cells from the VE were split into two clusters: the extra-embryonic VE (which corresponds to our exVE clusters) and another including cells from the embryonic VE, which includes the AVE. Hence, our first step consisted in the identification of a cluster of AVE cells at both the 5.5 and 6.5 dpc stages.

To this aim, we selected only embryonic VE cells and we found the intersection of the top 3,000 HVGs from our Smart-seq2 dataset (using only cells from our AVE and emVE clusters) at the corresponding stage with the genes quantified in the 10x data (Figures 5A, S4B, and S4C). This set of genes was used for all the analyses described below.

We computed the knn graph with Euclidean distance on the first 10 principal components with the default value for *k*, and we used the Leiden algorithm^94^ to cluster the cells (Scanpy function “sc.tl.leiden”). The resolution was fixed in such a way to obtain two clusters at each stage, which were annotated as AVE and emVE based on the expression of known markers.

As a next step, we searched for sub-populations of cells in the AVE and the exVE clusters at 6.5 dpc using the procedure explained below in detail. For each of these clusters, we performed Leiden clustering on the knn graph based on the Euclidean distance between the first 10 principal components computed on the top 2,000 HVGs. To estimate the number of clusters, we tried several combinations of the clustering parameters, i.e., the number of nearest neighbours k and the resolution *r*: we took *k* ∈ [15,35], with a step of 5, and *r* ∈ [0.1,1], with a step of 0.1. For each pair of (*k*,*r*) values, the robustness of clusters was tested with a gene sub-sampling procedure, with the same strategy described above. To mitigate the risk of overfitting, we kept only clusters that have several specific marker genes greater than 1; the clusters with 0 or 1 specific markers were merged with the closest of the remaining clusters, based on the relative Euclidean distances between the cluster centroids. We considered a marker gene as “specific” if (i) it has a log-normalized mean expression larger than 0.01 and (ii) it is statistically significantly upregulated in all the pairwise comparisons with the other clusters.

By doing so, we obtained three sub-clusters in the AVE and two sub-clusters in the exVE at 6.5 dpc, which were annotated on the basis of HCR experiments, as shown in Figure 5.

The same type of sub-clustering gave only a single cluster in 5.5 dpc AVE cells. Hence, we used a more supervised approach to verify whether the gene signature distinguishing AVE-medial and -lateral subclusters at 6.25 dpc can separate AVE sub-populations at 5.5 dpc. To this aim, we selected the top 10 upregulated and the top 10 downregulated genes between late-AVE-lateral and -medial cells at 6.25 dpc (Wilcoxon test with Benjamini-Hochberg correction method). Using these genes, we could identify two clusters in the AVE at 5.5 dpc with the Leiden algorithm (using *k*=15) (see Figure 5B).

#### Cell-cell communication analysis

The analysis of intercellular communication in the scRNA-seq data at 5.5 and 6.25 dpc has been performed using a curated list of 2,548 ligand-receptor pairs LRPs,^45^ to which we added the following LRPs based on previously reported direct interactions: CER1-MRC2,^95^ CER1-BMP2/BMP4,^96^ CER1-NODAL,^97^ LEFTY1-NODAL,^98^ LEFTY1-BMPR2.^99^ We selected only AVE, emVE and Epi cells, and we filtered out genes detected in fewer than 10 cells or with log-normalized mean expression computed on non-zero values smaller than 1.

Following the approach of Solovey and Scialdone,^46^ we represented each LRP α as a weighted directed graph, where the number of nodes *N* is equal to the number of cell types (i.e., in our case AVE, emVE and Epi). The elements of the adjacency matrix of the graph for the LRP are given by:

$$w_{ij}^{\alpha }=(\varphi_{i}^{\alpha }*\varphi_{j}^{\alpha })*\left(p_{i}^{\alpha }*p_{j}^{\alpha }\right)\text{,}$$

is the fraction of cells in the node *i* expressing the ligand and $\varphi_{j}^{\alpha }$ the fraction of cells in the node *j* expressing the receptor. All values of $\varphi_{i}^{\alpha }$ and $\varphi_{j}^{\alpha }$ smaller than 0.1 were set to 0. $p_{i}^{\alpha }={\underline{c}}_{i,\setminus 0}^{\alpha }/\max_{i}{\underline{c}}_{i,\setminus 0}^{\alpha }$, i.e., the ratio between the average of the non-zero log-expression values of the ligand in node *i* and the maximum of these averages computed over the different nodes. If the LRP includes a complex, $p_{i}^{\alpha }$ and $\varphi_{i}^{\alpha }$ are the product of the p and φ of the genes belonging to the complex, respectively.

We filtered (i.e., set to 0) weights smaller than the 50th percentile of the weight distribution computed on all LRPs, and we eliminated LRPs with a null weight matrix. We ended up with 413 and 471 LRPs at 5.5 and 6.25 dpc, respectively.

Next, we considered LRPs containing at least one of the genes upregulated in the AVE at both 5.5 and 6.25 dpc, as identified from the diffusion pseudotime analysis. This additional filtering resulted in 43 LRPs containing ‘high-in-AVE’ ligands or receptors (see Figure 6B). We used COMUNET^46^ to visualise the communication patterns, in particular those involving the high-in-AVE ephrins and Eph receptors (*Efna5* and *Ephb3*) (Figure S5F).

#### Analysis of genes in the Ephrin/Eph-signalling pathway

We considered genes in the Ephrin/Eph-signalling pathway and with log-normalized mean expression larger than 0.5, obtaining 14 genes at 5.5 dpc and 13 genes at 6.25 dpc. Using these genes, we performed a Principal Components Analysis at each stage, using the Scanpy function “scanpy.tl.pca”, as described above (Figures S5A–S5C). By doing so, we noticed that the five cell types in the dataset are separated at 6.25 dpc, while the result at 5.5 dpc is less clear.

Next, we asked which genes contributed significantly to the first three PCs at each stage. To this end, we generated 1,000 bootstrap samples of the cells and we performed a PCA on each of them. We assigned a *p*-value to each PC loading by computing the fraction of sign inversions obtained in the bootstrap procedure and accounting for possible axis reflections, as described by Peres-Neto et al.^100^ We considered genes as significant if they have at least one loading (considering the first three PCs) with *p*-value smaller than 0.01. We obtained 6 significant genes at 5.5 dpc and 8 at 6.25 dpc. The expression of the significant *Ephrin* genes at each stage is shown in the dot plots in Figure S5D.

#### In situ Hybridization Chain Reaction (HCR)

All the HCR probes were synthesised by Molecular Instruments (molecularinstruments.org, Pasadena, CA). *In situ* HCR v3.0 was carried out as previously described,^101^ with the following modifications: embryos were dissected in ice-cold M2 medium, before directly fixing in 4% PFA (SantaCruz, sc281692) overnight at 4°C; proteinase-treatment was done with 10 *μ*g/mL proteinase K (Thermo Scientific, EO0491), for 90 seconds at RT but using solutions pre-warmed to 37°C; post-fixation in 4% PFA was performed at 4°C for 20 minutes. When processing large number of embryos together, extra care was taken to make sure the temperature of the hybridisation and probe wash buffers the embryos were in didn’t drop below 37°C between transfers, by using prewarmed heat blocks. Methanol-dehydration/rehydration steps were omitted for samples requiring phalloidin-staining for F-actin visualisation. Following the HCR protocol, the samples were cleared in 87% (v/v) glycerol (Fisher Scientific, G/0650/17) in PBS (Sigma-Aldrich, P4417) for at least 3 days at 4°C before mounting for imaging using the same media.

#### Wholemount immunofluorescence

Embryos were isolated and staged as described above, and fixed in 4% PFA in PBS at RT for 30 minutes (or 5 minutes in ice-cold 1:1 acetone:methanol solution for Cytokeratin and Drebrin-1 staining), washed three times for 10 minutes in 0.1% Triton X-100 (Sigma-Aldrich, T8787) in PBS at RT; incubated in 0.25% Triton X-100 in PBS for 15 min at RT for permeabilization; washed twice for 5 minutes in 0.1% Tween-20 (Sigma-Aldrich, P1379) in PBS (PBST) at RT; blocked overnight in blocking reagent (5% donkey serum (Sigma-Aldrich, D9663), 3% bovine serum albumin (Sigma-Aldrich, A7906) in PBST) at 4°C; incubated overnight in primary antibodies diluted in the blocking reagent; washed three times for 10 minutes each, in PBST at RT; incubated overnight at 4°C in secondary antibodies and fluorophore-conjugated phalloidin diluted in PBST; washed thrice for 5 minutes in PBST at RT and the samples were cleared in depression slides with VECTASHIELD anti-fade mounting media containing DAPI (Vector Labs, H-1200) for 3 days at 4°C, before mounting for imaging using the same media.

The primary antibodies used were: 1:50 Rabbit anti-CK19 (Proteintech, 10712-1-AP), 1:100 Rabbit anti-Drebrin1 (Proteintech, 10260-1-AP), 1:100 Rabbit anti-MARCKS (Proteintech, 10004-2-Ig), 1:50 rabbit anti-MARCKSL1 (Proteintech, 10002-2-AP), 1:100 anti-Cytokeratin 8 (Proteintech, 10384-1-AP), 1:50 rabbit anti-phospho(Ser23)CK8 EP1629Y (Abcam, ab76584), 1:100 Goat anti-PlexinA1 (R&D Systems, AF4309), 1:200 Rabbit anti-Oct4 (Abcam, ab19657). The secondary antibodies used were: Alexa Flour (AF)-555 Donkey anti-rabbit IgG (Invitrogen, A31572) and AF-633 Donkey anti-goat IgG, AF-488 (Invitrogen, A21082) both at 1:200. For F-actin staining either Phalloidin-Atto 550 (Sigma-Aldrich, 19083) or Phalloidin-Atto 647N (Sigma-Aldrich, 65906) were used at a final concentration of 1 mg/mL.

#### Embryo culture and inhibitor studies

Embryo culture media made up of 49.5% CMRL-1066 (Pan BioTech, P04-84600), 49.5% KnockOut serum-replacement (Gibco, 10828-010), supplemented with was 1% L-glutamine was pre-equilibrated at 37°C and 5% CO_2_ for at least 2 hours prior to use. *Hhex-GFP* transgenic embryos dissected at 5.5 dpc were briefly screened on a 35 mm glass bottom dish (MatTek Life Sciences, P35G-1.5-14-C) using low laser intensities under a confocal microscope to select those embryos where the GFP-positive AVE cells were still at the distal tip. The ectoplacental cone was left on the embryos to facilitate survival during culture. The embryos were cultured in Nunc™ Lab-Tek™ II chambered coverglass slides (Thermo Fisher, 155409) with 400μl of embryo culture media per chamber, under control- and treated-conditions at 37°C and 5% CO_2_. Culture periods from 4 to 15 hours were tested, but a duration of 4 hours was sufficient to document the complete migration of the AVE from the distal tip to the epiblast–ExE boundary and laterally across the embryo. To perturb Ephrin/Eph-signalling, the small molecule inhibitor NVP-BHG712 (Selleck Chemicals, A8683) was used at a final concentration of 100*μ*M and an equal volume of DMSO was used in the controls. To block Semaphorin-signalling, Mouse anti-Semaphorin6D (Santa Cruz Biotechnology, sc393258) or Goat anti-PlexinA1 (R&D Systems, AF4309) were used at a final concentration of 10 *μ*g/mL.

#### Image acquisition, live-imaging and processing

Fixed-samples were imaged on a ZEISS LSM 880 confocal microscope using 20x/0.75, 40x/1.2 W Korr M27 water immersion or Plan-Apochromat 63x/1.4 OIL DIC M27 objective as appropriate. For super-resolution imaging to visualise subcellular localisation an Airyscan detector (Zeiss) was used. Z-stacks of embryos were acquired at 1*μ*m interval using non-saturating scan parameters. Opacity rendering as 3D volumes and videos were made using the Volocity Software (Improvisions). For HCR and immunofluorescence images, the 3D renderings are presented as surface renderings or maximum intensity projections (as indicated in figure legends). Figures were prepared with Adobe Photoshop 2020 and Adobe Illustrator 2020 (Adobe Inc.). Individual 3D opacity renderings for each channel were combined from individual layers to create merged images. For the quantification of the HCR signals, regions of interest (ROIs) for anterior and posterior regions (epiblast or VE) were drawn on the mid-sagittal section of each embryo using the Fiji ImageJ software,^102^ using the DAPI channel for reference. To account for the differences in overall intensity between embryos, the mean intensities of the *Wnt3* and *Cer1* signals in the anterior and posterior were individually normalised against the DAPI signal within the ROI, and the anterior/posterior expression ratio was calculated.

For live-imaging, embryos were dissected as detailed above and secured in position in between pulled glass capillaries in an 8-Well μ-Slide #1.5 (Ibidi, IB-80807) sample carrier containing 400μl of pre-equilibrated embryo culture medium (see above) per well. The embryos were cultured at 37°C and 5% CO2 for up to 9 hours, and during this time imaged on a ZEISS Laser Scanning 880 confocal microscope (LSM) using the 40x/1.2 objective or on a ZEISS Lattice Lightsheet 7 microscope (LLSM) using 10x/0.4 water immersion illumination objectives, 48x/1.0 detection objectives and a pre-defined 100*μ*m x 1800*μ*m Sinc3 lightsheet beams. Images were acquired at 6 (LSM) or 5 (LLSM) minute time resolution respectively. The LSM data was exported as.tiff stacks for MOSES analysis. The LLSM data was deskewed with coverglass correction and cropped using the ZEN Blue software (Zeiss), and the maximum length of the projections were measured using the line tool on Fiji Image J on the time frame where the maximum length for each projection was observed.

#### MOSES analysis

Control and mutant videos with Hhex-GFP labelled cells were rotated to be *en face* in Volocity Software (Improvisions) and maximum intensity projected to 2D timelapse videos of the surface. Videos were then temporally registered to remove artefactual motion due to drift and growth. This was done in two steps using the “Linear Stack Alignment with SIFT” plugin in Fiji ImageJ.^102^ In step 1 the raw videos were registered using ‘rigid’ as the expected transform type to remove rotation and translation by drift. In step 2 the rigid registered videos were re-registered using ‘similarity’ as the expected transform type to remove additionally scale due to growth. Default parameters were used for registration except for embryos with low intensity which causes many erroneous matchings across frames. In these cases, we reduced the maximal alignment error to 10 pixels and closest/next closest ratio to 0.82 to both increase the number of interest points for matching and force the matching criteria to be more stringent. We then used Motion sensing superpixels (MOSES^52^) to characterise Hhex-GFP cell migration in the registered videos based on superpixel region-of-interest long-time tracking. MOSES was run with Farnebäck optical flow^103^ and dense tracking with an initial 1000 superpixels. In dense tracking, MOSES automatically seeds new superpixels during tracking to maintain uniform coverage over the field-of-view.^53^ This allows the emergence of new cells not present in the initial frame to be tracked. Each video was tracked both forwards (first frame to last) and in reverse (last to first frame) in time. Tracking forwards better captures where motion flows towards (i.e. motion sinks) whereas reverse tracking better captures where motion originates from (i.e. motion sources). Each track was joined together with its neighbour tracks to form a mesh – the MOSES mesh, whereby a neighbour is defined as being a distance < 1.2 times mean superpixel width at the starting frame of tracking. From the MOSES mesh, we computed the motion saliency map to visualize local motion sources and sinks.^52^^,^^53^ We then computed the mesh strain curve, defined as the mean absolute difference in mesh edge lengths relative to the undeformed mesh edge lengths over all superpixel tracks for each time frame. The result is a 1D motion signature the same length as the number of frames in the video that summarizes the mesh dynamics and is a 1D vector suitable for clustering and classification analysis. The motion signatures were extracted for all videos and truncated to the minimum frame number over all videos (90 frames) to enable analysis over a common temporal duration (540 minutes). Finally, principal components analysis was applied to the concatenated motion signature from forward and backward tracking to unsupervised cluster the control and mutant videos. The loadings of PC1 and PC2 from the PCA are shown in Figures 6H′ and 6H″ respectively.

### Quantification and statistical analysis

Specific quantification parameters and statistical analyses used are described in detail under each method above and in the figure legends where necessary. The results of the statistical tests are presented in the figure legends, where ‘N’ always represents the number of embryos, unless stated otherwise (eg: cells, projections).

## Supplemental information

Document S1. Figures S1–S6 and supplemental reference

Table S1. Top 50 marker genes per cluster at 5.5 dpc, related Figure 1

Table S2. Top 50 marker genes per cluster at 6.25 dpc, related to Figure 1

Table S3. Lists of genes differentially expressed in diffusion pseudotime analysis of early-AVE and emVE cells at 5.5 dpc, related to Figure 1

Table S4. Lists of genes differentially expressed in diffusion pseudotime analysis of late-AVE and emVE cells at 6.25 dpc, related to Figure 1

Table S5. Lists of proteins upregulated in abembryonic half, among those differentially expressed between abembryonic and embryonic halves in the proteomics dataset, related to Figure 2

Table S6. Lists of proteins upregulated in the embryonic half, among those differentially expressed between abembryonic and embryonic halves in the proteomics dataset, related to Figure 2

Table S7. Lists of proteins upregulated in abembryonic half, among those differentially expressed between abembryonic and embryonic halves in the phosphoproteomics dataset, related to Figure 2

Table S8. Lists of proteins upregulated in the embryonic half, among those differentially expressed between abembryonic and embryonic halves in the phosphoproteomics dataset, related to Figure 2

Table S9. Lists of specific marker genes of the late-AVE sub-clusters at 6.25 dpc, related to Figure 5

Table S10. Lists of differentially expressed genes between the late-AVE transitional sub-cluster and the emVE cluster at 6.25 dpc, related to Figure 5

Table S11. Lists of differentially expressed genes between the late-AVE-lateral and late-AVE-medial sub-clusters at 6.25 dpc, related to Figure 5

Table S12. Lists of differentially expressed genes between exVE-distal and exVE-proximal sub-clusters at 6.25 dpc, related to Figure 5

Table S13. List of genes showing differential isoform expression between the early-AVE vs. emVE and late-AVE vs. emVE at 5.5 and 6.25 dpc, respectively, related to Figure 1

Table S14. Raw data for the proteomics experiments at 6.25–6.5 dpc, related to Figure 5

Table S15. Raw data for the phosphoproteomics experiment at 6.25–6.5 dpc, related to Figure 5

Table S16. Results of the functional enrichment analysis on the proteomics data listing enriched terms in the embryonic and abembryonic halves at 6.25–6.5 dpc, related to Figure 5

Table S17. Results of the functional enrichment analysis on the phosphoproteomics data listing enriched terms in the embryonic and abembryonic halves at 6.25–6.5 dpc, related to Figure 5

Document S2. Article plus supplemental information
