# Supplementary material for: An integrated approach identifies the molecular underpinnings of murine anterior visceral endoderm migration
Source: Dev Cell. 2024 Sep 9;59(17):2347–2363.e9. doi: 10.1016/j.devcel.2024.05.014 (PMC11511681; doi:10.1016/j.devcel.2024.05.014)
Supplement: Document S1. Figures S1–S6 and supplemental reference [file mmc1.pdf]

Developmental Cell, Volume *59*

Supplemental information

An integrated approach identifies the molecular underpinnings of murine anterior visceral endoderm migration

Shifaan Thowfeequ, Jonathan Fiorentino, Di Hu, Maria Solovey, Sharon Ruane, Maria Whitehead, Felix Zhou, Jonathan Godwin, Yentel Mateo-Otero, Bart Vanhaesebroeck, Antonio Scialdone, and Shankar Srinivas

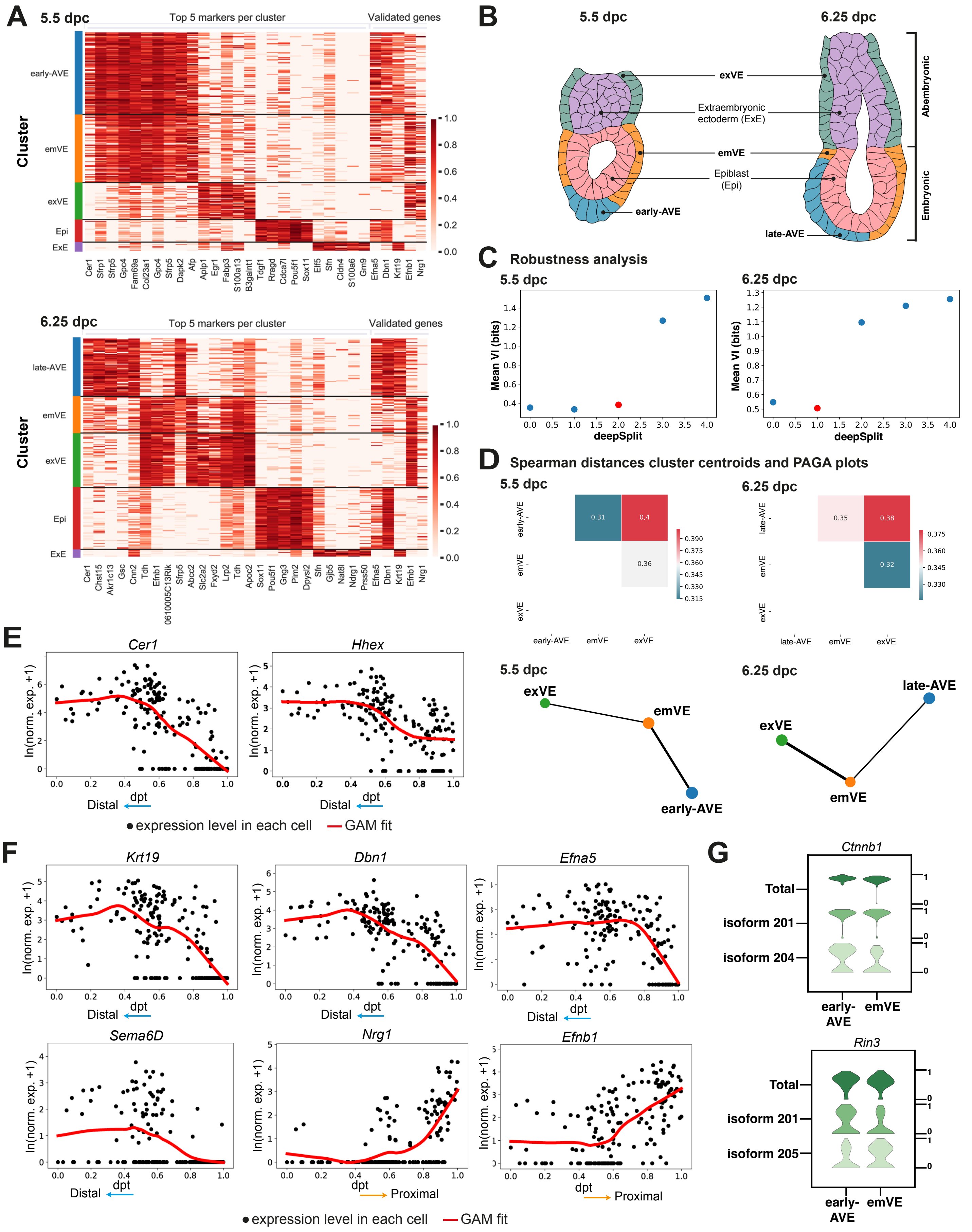

Supplementary Figure S1

(see next page for figure legend)

Supplementary Figure S1. Expression of newly computed marker genes, robustness analysis for cell clustering, similarity for visceral endoderm clusters and expression in pseudotime of selected 'high-in-AVE' genes.

(A) Heatmaps showing the expression of the top 5 marker genes per cluster that we computed, at 5.5 dpc (top) and 6.25 dpc (bottom), and of the genes that have been selected for experimental validation (*Efna5*, *Dbn1*, *Krt19*, *Efnb1*, *Nrg1*). The normalized log expression levels of each gene are standardised so that they vary within the interval [0,1].

(B) Illustrations of mid-sagittal sections through 5.5 and 6.25 dpc embryos outlining the nomenclature used in this study to describe the different cell-types of the embryo at these stages.

(C) Plot of the mean Variation of Information (VI) as a function of the deepSplit parameter, used for choosing the number of clusters in the clustering of the scRNA-seq data at 5.5 dpc (left) and 6.25 dpc (right). The red dot indicates the value of deepSplit chosen at each stage (see Methods for the details regarding the robustness analysis for cell clustering).

(D) Matrix plots of the Spearman distances between the centroids of the AVE, emVE and exVE clusters, at 5.5 dpc (left) and 6.25 dpc (right), and partition-based graph abstraction (PAGA) plots for the AVE, emVE and exVE clusters, at 5.5 dpc (left) and 6.25 dpc (right).

(E) Expression patterns of known AVE markers (*Cer1*, *Hhex*) at 5.5 dpc in the early-AVE and emVE clusters, as a function of diffusion pseudotime.

(F) Expression patterns of selected genes from the 'high-in-AVE' group (*Efna5*, *Krt19*, *Sema6d*, *Dbn1*) and low-in-AVE group (*Efnb1, Nrg1*) at 5.5 dpc in the early-AVE and emVE clusters, as a function of diffusion pseudotime.

(G) Violin plots showing the log expression of total transcripts (top row) and selected isoforms for *Ctnnb1* and *Rin3,* scaled by the maximum value in the early-AVE and emVE clusters at 5.5 dpc. Isoform numbers refer to Ensembl nomenclature.

Related to Figure 1.

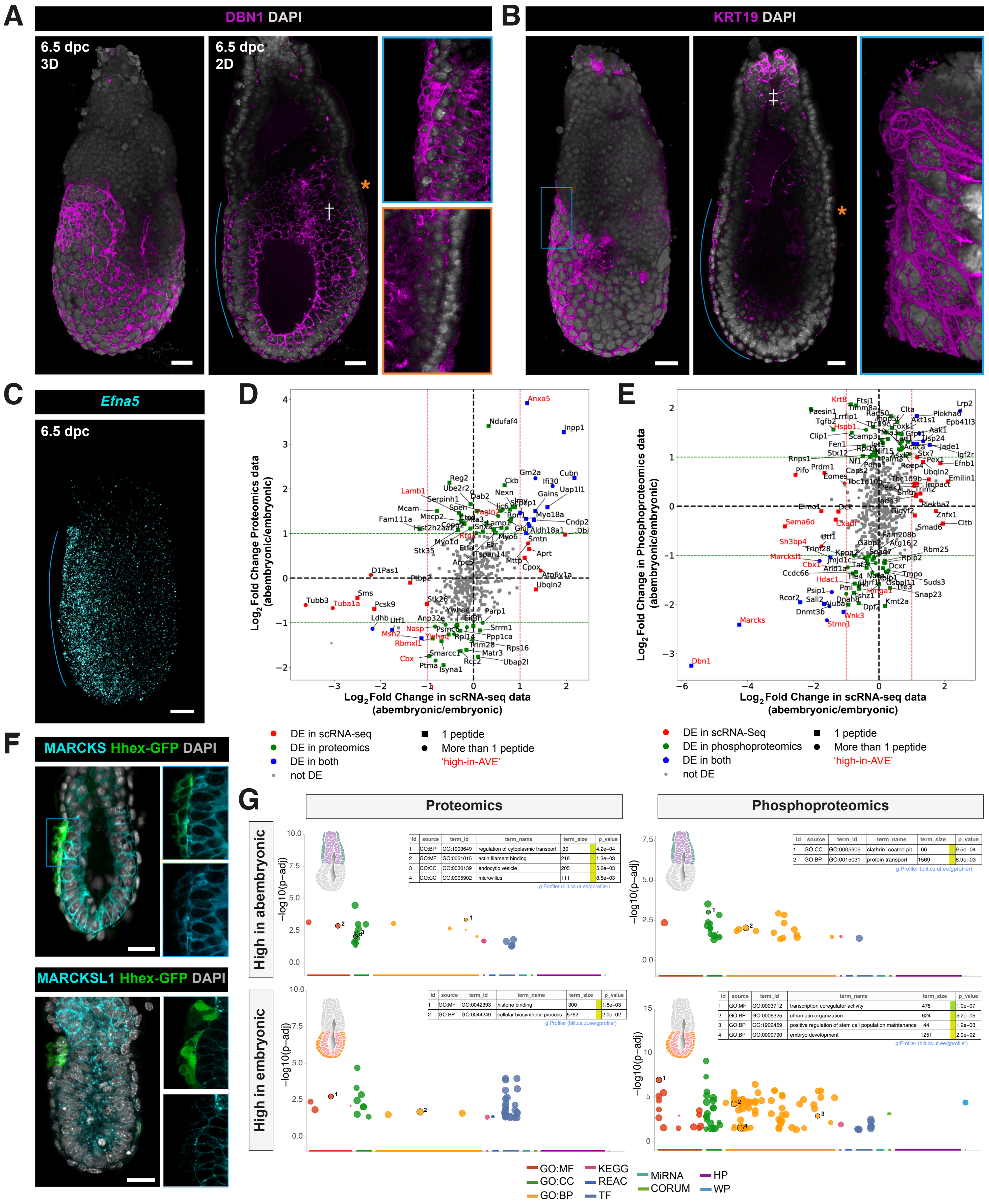

Supplementary Figure S2

(see next page for figure legend)

Supplementary Figure S2. Analysis of proteomics and phosphoproteomics from mouse embryos at 6.25-6.5 dpc and comparison with scRNA-seq data

(A-C) Continued anterior-enriched expression of ‘high-in-AVE’ markers DBN1 and KRT19 (visualised by immunofluorescence; 3D rendering and 2D mid-sagittal section) and *Efna5* (visualised by HCR; 3D rendering) at 6.5 dpc. The blue lines indicate the position of the AVE, and the orange asterisks mark the posterior side of the embryos. † and ‡ mark the expression of DBN1 and KRT19 in the epiblast and ExE respectively.

(D-E) Scatter plot of $\log_2$ Fold Change, computed from a differential expression analysis between the EXE and the EPI halves, in proteomics vs scRNA-seq data (D) and phosphoproteomics vs scRNA-seq (E) from 6.25-6.5 dpc embryos (see Methods). Each marker represents a protein/gene, the different colours indicate whether the protein is differentially expressed only in the scRNA-seq (red), only in the proteomics or phosphoproteomics (green) or in both datasets being compared (blue), the different markers indicate whether a protein has only one peptide (square) or if it has more than one peptide (circle). The names of proteins corresponding to genes from the ‘high-in-AVE’ group are highlighted in red. For D, Pearson’s correlation coefficient=0.52; p-value=8.5×10^{-33} and for E, Pearson’s correlation coefficient=0.39; p-value=1.0×10^{-19}.

(F) Mid-sagittal sections of 6.5 dpc embryos showing the expression of MARCKS (N=4) and MARCKSL1 (N=6). Hhex-GFP expression (green) marks the AVE. The right panels show super-resolution Airyscan images of the boxed AVE region to highlight specific subcellular localisation; cytosolic expression in the AVE for MARCKSL1 and membrane-associated expression in the AVE for MARCKS, corresponding to their phosphorylated and unphosphorylated forms respectively.

(G) Manhattan plots and tables of selected significant terms from a functional enrichment analysis performed on the proteins upregulated in the embryonic or abembryonic half, for the proteomics and phosphoproteomics datasets.

Scale=20μm and all embryos are orientated with the anterior on the left.

Related to Figure 2.

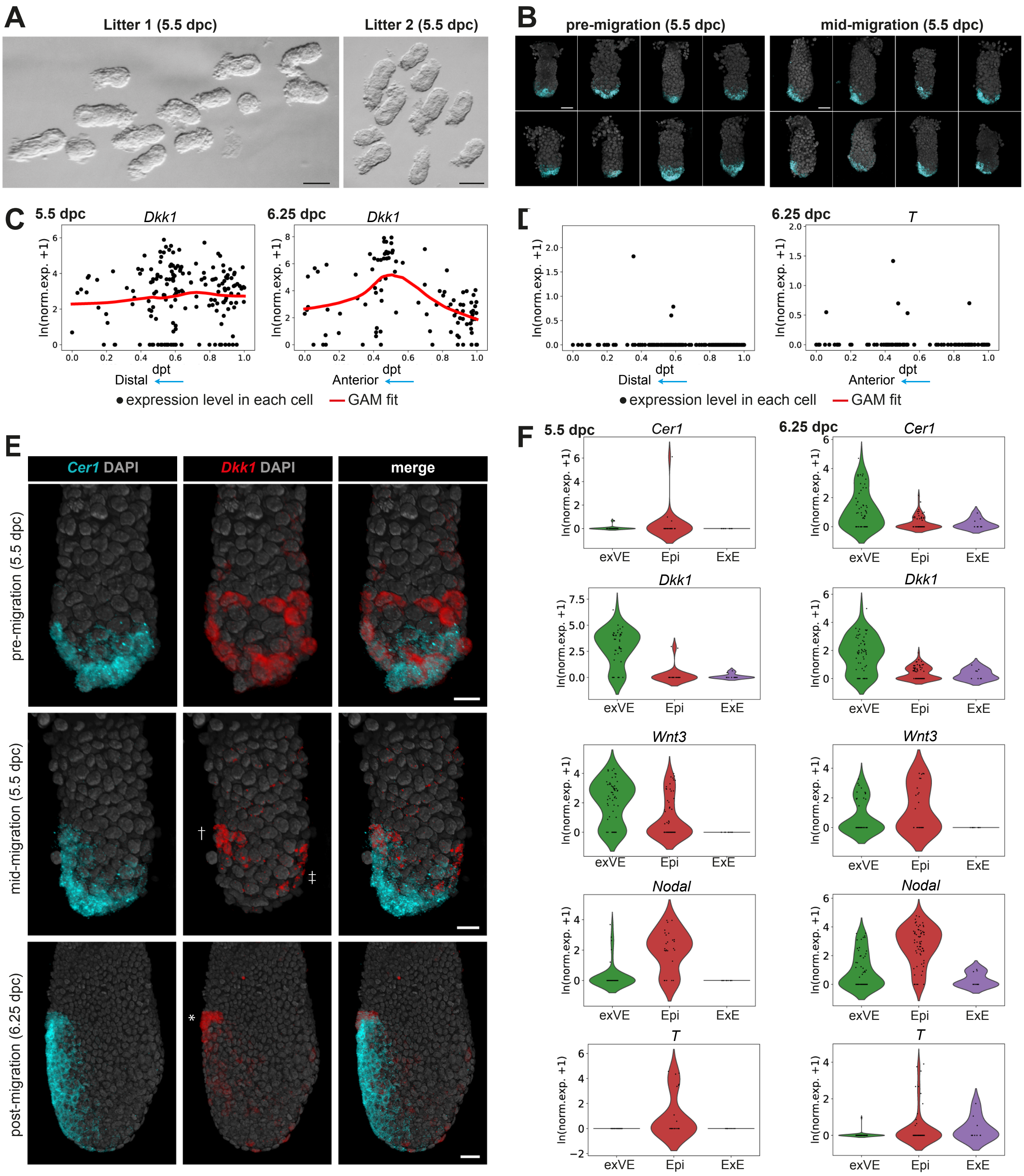

Supplementary Figure S3

(see next page for figure legend)

Supplementary Figure S3. Expresson of key anterior–posterior symmetry breaking markers in different cell population of 5.5 and 6.25 dpc embryos

(A) Intra- and inter-litter variation in the size and morphology of embryos isolated at 5.5 dpc. Scale=100μm.

(B) Variation in size and morphology of 5.5 dpc embryos classified as 'pre-migration' and 'mid-migration' (based on the position of the AVE) showing the corresponding expression of *Cer1* for each embryo. Scale=50μm.

(C) Expression pattern of *Dkk1* in the AVE and emVE clusters, as a function of diffusion pseudotime, at 5.5 dpc (left) ad 6.25 dpc (right).

(D) Volume renderings showing the changes to the *Dkk1* expression domain (visualised by HCR) relative to the position of the *Cer1*-expressing AVE cells as they migrate from the distal portion of the embryo to the epiblast-ExE boundary. † and ‡ mark the segregated anterior and posterior expression domains of *Dkk1* at mid-migration stages before it gets confined to the leading (most-proximal) cells of the AVE (*) at 6.25 dpc. Scale=20μm and all embryos are orientated with the anterior on the left.

(E) Expression patterns of *T* in the AVE and emVE clusters, as a function of diffusion pseudotime, at 5.5 dpc (left) and 6.25 dpc (right).

(F) Violin plots showing the normalised log expression levels of *Cer1*, *Dkk1*, *Wnt3*, *Nodal* and *T* in the exVE, Epi and ExE clusters.

Related to Figures 1 and 3.

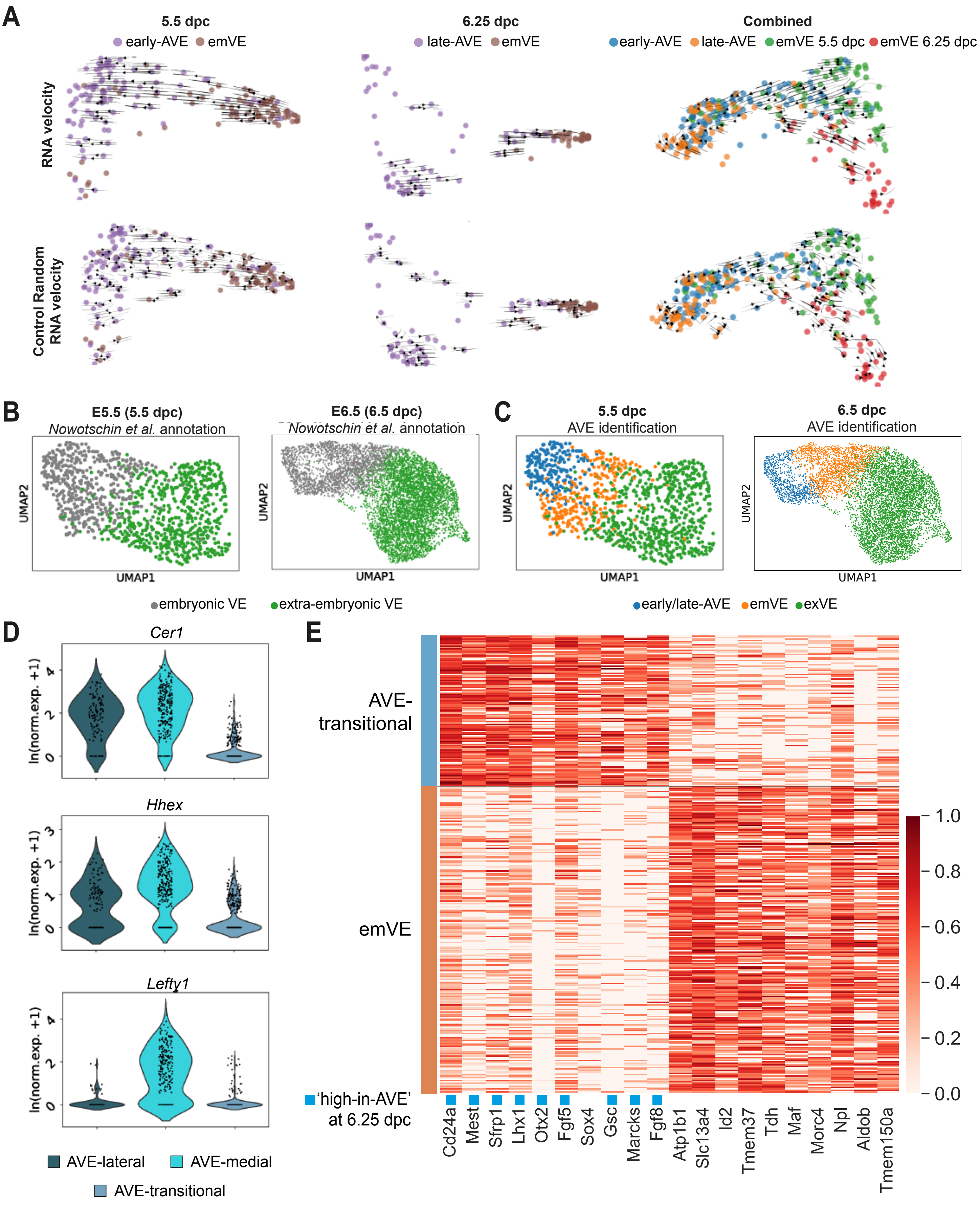

Supplementary Figure S4

(see next page for figure legend)

Supplementary Figure S4. Changing RNA velocity streams for 5.5 and 6.25 dpc VE cells and characterization of the AVE-transitional cell population

(A) RNA velocity streams projected on the first two diffusion components of a diffusion map (top row) computed on AVE and emVE cells at 5.5 dpc (top left), 6.25 dpc (top centre) and on the combined data from the two stages (top right). The bottom row shows the projection on the same embeddings of randomized RNA velocities, as a control.

(B) UMAP plots of the cells annotated as visceral endoderm in Nowotschin et al.[S1], at 5.5 dpc (left) and 6.5 dpc (right). Cells are coloured according to the original annotation as embryonic VE and extra-embryonic VE.

(C) Same UMAP plots as in panel (B), but with the cells coloured according to our annotation (early/late-AVE, emVE and exVE clusters), obtained as described in Figure 5A and in the Methods.

(D) Violin plots showing the normalized log expression levels of *Cer1*, *Hhex* and *Lefty1* in the late-AVE sub-clusters (AVE-lateral, AVE-medial and AVE-transitional) at 6.5 dpc.

(E) Heatmap showing the expression of the top 10 genes upregulated in the AVE-transitional sub-cluster and the top 10 genes upregulated in the emVE cluster, obtained through a differential expression analysis between the AVE-transitional and the emVE clusters at 6.5 dpc. The normalized log expression levels of each gene are standardised so that they vary within the interval [0,1]. Genes that also belong to the 'high-in-AVE' group we identified at 6.25 dpc are flagged in blue.

Related to Figures 4 and 5.

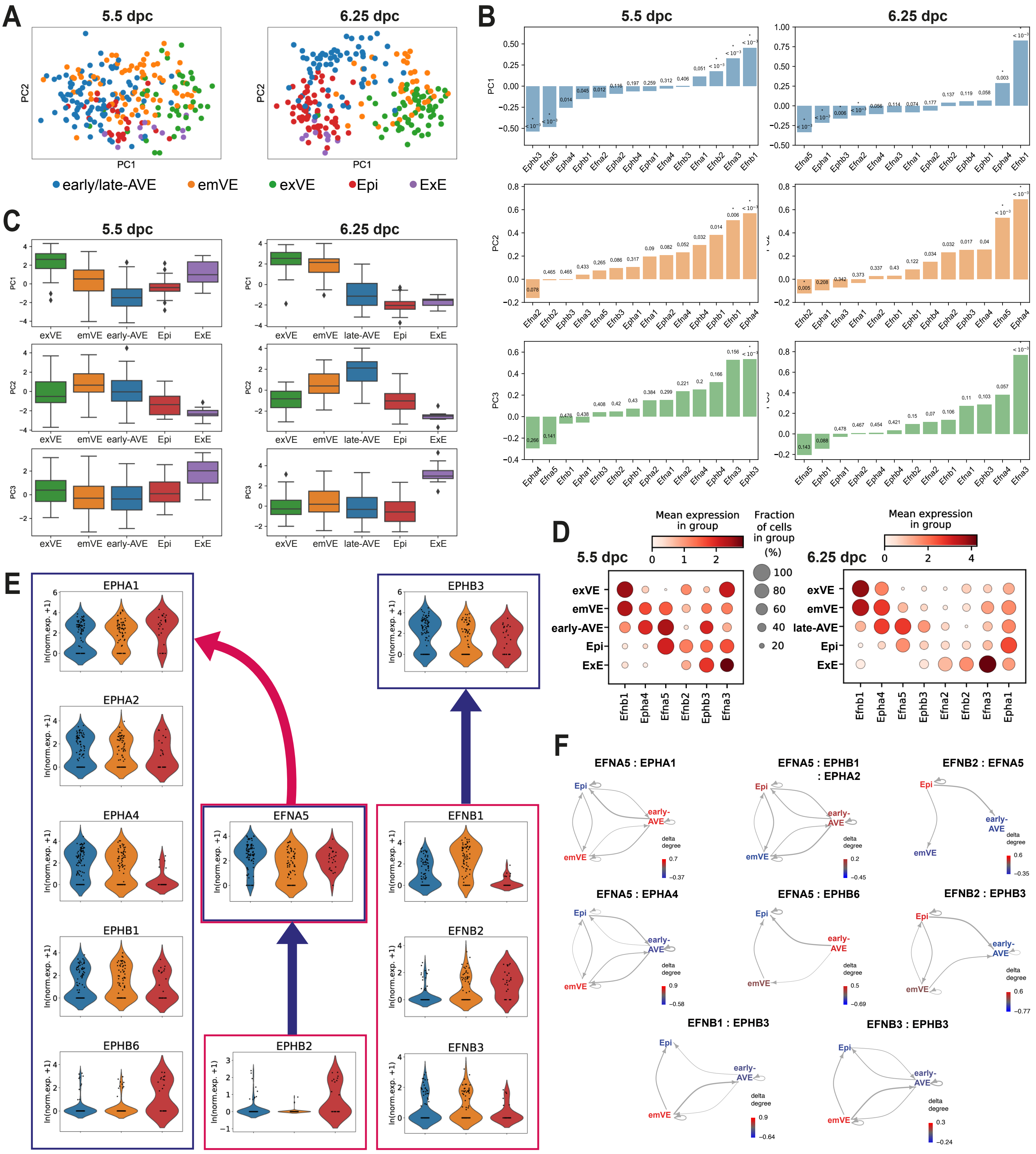

Supplementary Figure S5

(see next page for figure legend)

Supplementary Figure S5. Cell-type specific expression and intercellular communication patterns of genes belonging to the Ephrin/Eph signalling pathway

(A) Plots showing the first two principal components of a principal components analysis (PCA) performed on genes belonging to just the Ephrin/Eph-signalling pathway, at 5.5 dpc (left) and 6.25 dpc (right).

(B) Loadings of the first three principal components for the genes used in the PCA, at 5.5 dpc (left) and 6.25 dpc (right). The p-values indicated for each gene were computed using a bootstrap procedure (*see* Methods). Significant genes (p-value < 0.01) are marked (*) in the loading plots. We obtained 6 and 8 significant genes at 5.5 and 6.25 dpc, respectively.

(C) Box plots of the values of the first three principal components grouped by cluster, at 5.5 dpc (top) and 6.25 dpc (bottom).

(D) Dot plots of genes belonging to the Ephrin/Eph signalling pathway at 5.5 dpc (top) and 6.25 dpc (bottom). The genes were selected as those showing a significant contribution to the first three principal components in a Principal Components Analysis performed on genes belonging to the Ephrin/Eph signalling pathway (see Methods).

(E) Violin plots showing the normalized log expression levels of the genes belonging to the Ephrin/Eph-signalling pathway shown in Figure 6A, at 5.5 dpc. The red arrow shows that EFNA5 acts as a ligand for the receptors EPHA1, EPHA2, EPHA4, EPHB1, and EPHB6, while the blue arrows show that EFNA5 is a receptor for EPHB2 due to the bidirectional nature of signalling within this pathway. EPHB3 is a receptor of the ligands EFNB1, EFNB2, EFNB3.

(F) Intercellular communication patterns for the LRPs involving EFNA5 and EPHB3 encoded by genes belonging to the ‘high-in-AVE’ group at 5.5 dpc.

Related to Figure 6.

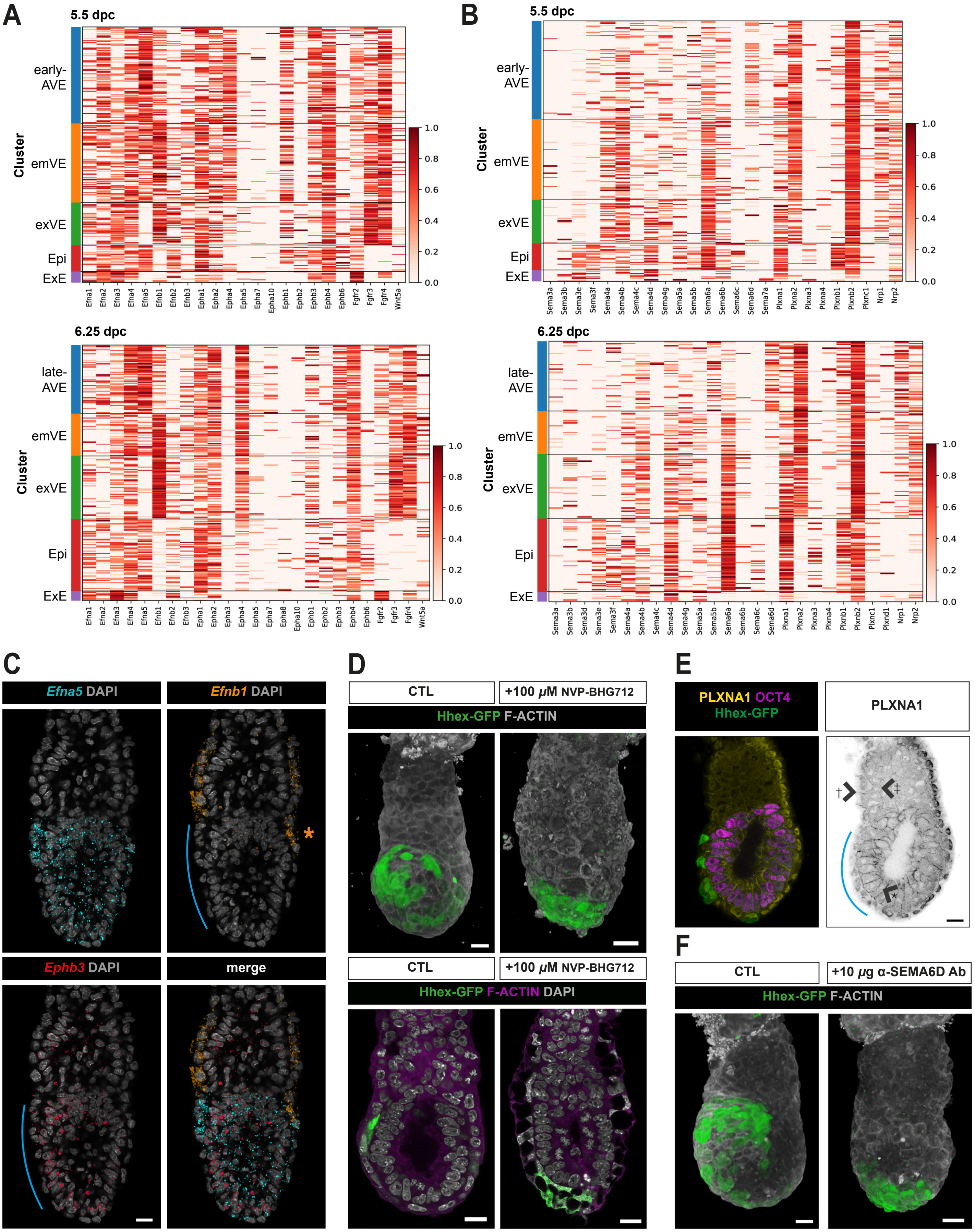

Supplementary Figure S6

(see next page for figure legend)

Supplementary Figure S6. Expression of members of Ephrin- and Semaphorin-signalling pathways in the peri-implantation embryo and the effect of their pharmacological blockade on AVE migration

(A) Heatmap showing the expression of genes encoding components of the Ephrin/Eph-signalling pathway, at 5.5 dpc (left) and 6.25 dpc (right). The normalized log expression levels of each gene are standardised so that they vary within the interval [0,1].

(B) Heatmap showing the expression of genes encoding components of the Semaphorin/Plexin-signalling pathway, at 5.5 dpc (left) and 6.25 dpc (right). The normalized log expression levels of each gene are standardised so that they vary within the interval [0,1].

(C) Optical sections though late 5.5 dpc embryos showing the expression of anterior-enriched *Efna5* and *Ephb3* relative to that of the posterior-enriched *Efnb1* (visualised with HCR). The blue line indicates the extent of the AVE and the orange asterisk marks the posterior side of the embryo.

(D) Visualisation of AVE migration in embryos expressing the Hhex-GFP transgene reporter, cultured with (+100 μM NVP-BHG712) and without (CTL) the presence of a Ephrin/Eph-signalling inhibitor. The two images on the top are volume renderings showing the overall position of the AVE at the end of the culture period; the two images at the bottom are optical sections through the embryos showing the effects of the inhibitor on other cell types of the embryo including the emVE, exVE, and epiblast.

(E) Optical section through a late 5.5 dpc embryo showing the expression of PlexinA1 (PLXNA1- the receptor for the AVE-specific Semaphorin, SEMA6D) by immunofluorescence, in the membranes of epiblast (*), exVE (†) and ExE (‡). Hhex-GFP and OCT4 mark AVE and epiblast cells respectively. The blue lines show the position of the AVE.

(F) Volume renderings visualising AVE migration in embryos expressing the Hhex-GFP transgene reporter, cultured in the presence of an antibodies against SEMA6D (α-SEMA6D Ab) in comparison to a control (CTL) embryo.

Scale=20μm and all embryos are orientated with the anterior on the left.

Related to Figure 6.

SUPPLEMENTAL REFERENCES

[S1]. Nowotschin, S., Setty, M., Kuo, Y.-Y., Liu, V., Garg, V., Sharma, R., Simon, C.S., Saiz, N., Gardner, R., Boutet, S.C., et al. (2019). The emergent landscape of the mouse gut endoderm at single-cell resolution. Nature *569*, 361-367. 10.1038/s41586-019-1127-1.
